# Quantum-Dot-Based Scanning Probe d‑Band Hole Spectroscopy of Plasmonic Nanoparticles

**DOI:** 10.1021/acs.nanolett.6c03990

**Published:** 2026-10-01

**Authors:** Florian Küstner, Harald Ditlbacher, Andreas Hohenau, Dmitry N. Dirin, Maksym Kovalenko, Joachim R. Krenn

**Affiliations:** † Institute of Physics, 27267 University of Graz, 8010 Graz, Austria; ‡ Institute of Inorganic Chemistry, Department of Chemistry and Applied Biosciences, 31064 ETH Zürich, 8093 Zürich, Switzerland; ¶ Empa − Swiss Federal Laboratories for Materials Science and Technology, Laboratory for Thin Films and Photovoltaics, 8600 Dübendorf, Switzerland

**Keywords:** plasmon, hot carrier generation, colloidal quantum dots, Schottky barrier, photocurrent

## Abstract

Despite intense research efforts, quantitative energy-resolved measurements on hot carriers generated due to plasmon decay in metal nanoparticles are rare. This concerns in particular excitations below the Fermi level, i.e., holes. Here, we use a metallized scanning probe tip to measure photocurrents from plasmonic gold nanoparticles through a monolayer of colloidal lead sulfide quantum dots. Separating out current contributions from quantum dot excitons and thermionic emission, we achieve quantitative measurements on the hole excitation quantum efficiency in the gold nanoparticles in the 1.9–2.6 eV energy range. Therefore, we combine systematic experimental bias and wavelength variations and a comparison with the case of aluminum with extensive modeling. The detection scheme can be extended to electron currents and other energy ranges by simply using quantum dots with different energy levels.

## Graphical abstract

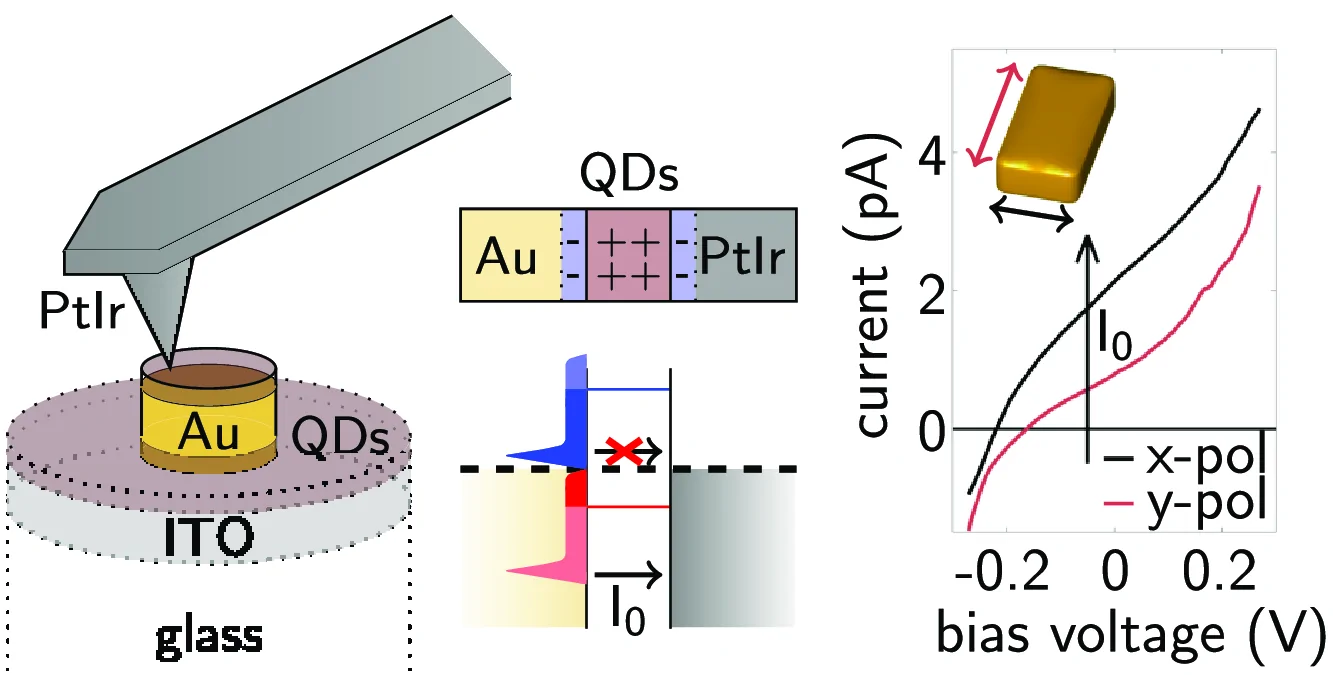

The nonradiative decay of surface plasmons in metals can generate energetic, nonequilibrum (*hot*) carriers that offer new opportunities for photocatalysis, photodetection and solar energy conversion.^1–4^ When plasmons are excited in metal nanoparticles, these bring along particularly strong absorption with spectral and spatial characteristics that are tunable by the particle geometry^5^ and that can thus be adapted to the respective application. Recent advances in nanostructure design, simulation and experiment have significantly widened our understanding of hot carriers.^6–8^ Experimentally, ultrafast pump-probe techniques and energy-resolved photoemission measurements have been used to investigate hot charge populations and their ultrafast dynamics.^9,10^

Photocurrent measurements were applied, e.g., to Schottky barriers between plasmonic nanoparticles and bulk silicon, evidencing the plasmon origin of the hot electrons.^11^ To some extent, electron energy discrimination was achieved by comparing Schottky and Ohmic contacts.^12^ The respective roles of intraband and interband excitations were analyzed^13^ and both, electron and hole photocurrents from copper nanostructures were detected on n- and p-type bulk GaN, respectively. The energy distribution of both carrier types has been analyzed in a scanning-probe-based molecular junction on a plasmon-carrying gold film.^14^ The hole current from gold nanoparticles on p-type GaN was spatially resolved with a conductive AFM tip.^15^ In any case, measurements providing high spatial and quantitative energy resolution are still rare.^10^

Among all molecular and semiconducting charge-acceptors to be combined with a plasmonic metal, semiconductor nanoparticles (colloidal quantum dots, QDs) appear particularly appealing, as they offer both chemical stability and straightforward energy-level tunability. In recent studies, we showed that a lead suflide (PbS) QD monolayer between a metal substrate and a metallized scanning probe tip forms a double Schottky junction with a built-in potential given by the work function difference of the electrodes, i.e., substrate and tip.^16,17^ Since the QD layer thus acts merely as a barrier for charge separation between two electrodes that set the voltage drop, a wide variety of QDs can be considered for such measurements, with potentially different energy levels allowing for carrier energy discrimination. PbS QDs are versatile candidates since they are stable and can be synthesized with size-dependent bandgaps in the 0.6–1.7 eV energy range.^18–20^ An ultrathin MAPbI_3_ ligand shell enables a measurable conductance and enables the fabrication of high-quality monolayers.^21^ In this Letter, we investigate the Schottky junction of a PbS/MAPbI_3_ QD monolayer on plasmonic gold and aluminum nanoparticles. The photocurrent across the QD layer is detected with high spatial resolution with the platinum iridium (PtIr)-coated tip of an atomic force microscope (AFM).

The current contributions from excitations within the QDs and those in the plasmonic particles are distinguished by systematically varying the bias and the illumination intensity. Comparing the effects due to the different electronic structure in gold and aluminum gives insight into hot charge excitation and transport. By varying the wavelength of the excitation light, we deduce quantitatively the quantum efficiency of hot hole excitation in gold in the 1.9–2.6 eV energy range, using PbS QDs with a diameter of 3.2 nm.

Single gold and aluminum nanoparticles were fabricated by electron beam lithography on thin glass slides covered by indium tin oxide (ITO), see Figure 1(a) and the Methods file of the Supporting Information for details. The 30 nm high gold particles have either a rectangular in-plane cross section with long and short axis lengths of 150 and 60 nm, respectively, (rectangular cuboid) or a square section with a side length of 60 nm (square cuboid). The disk-shaped aluminum particles have a diameter of 120 nm and a height of 20 nm. For optical extinction spectroscopy, extended arrays with a square pitch of 300 nm of nominally identical particles were fabricated.

**Figure 1:**
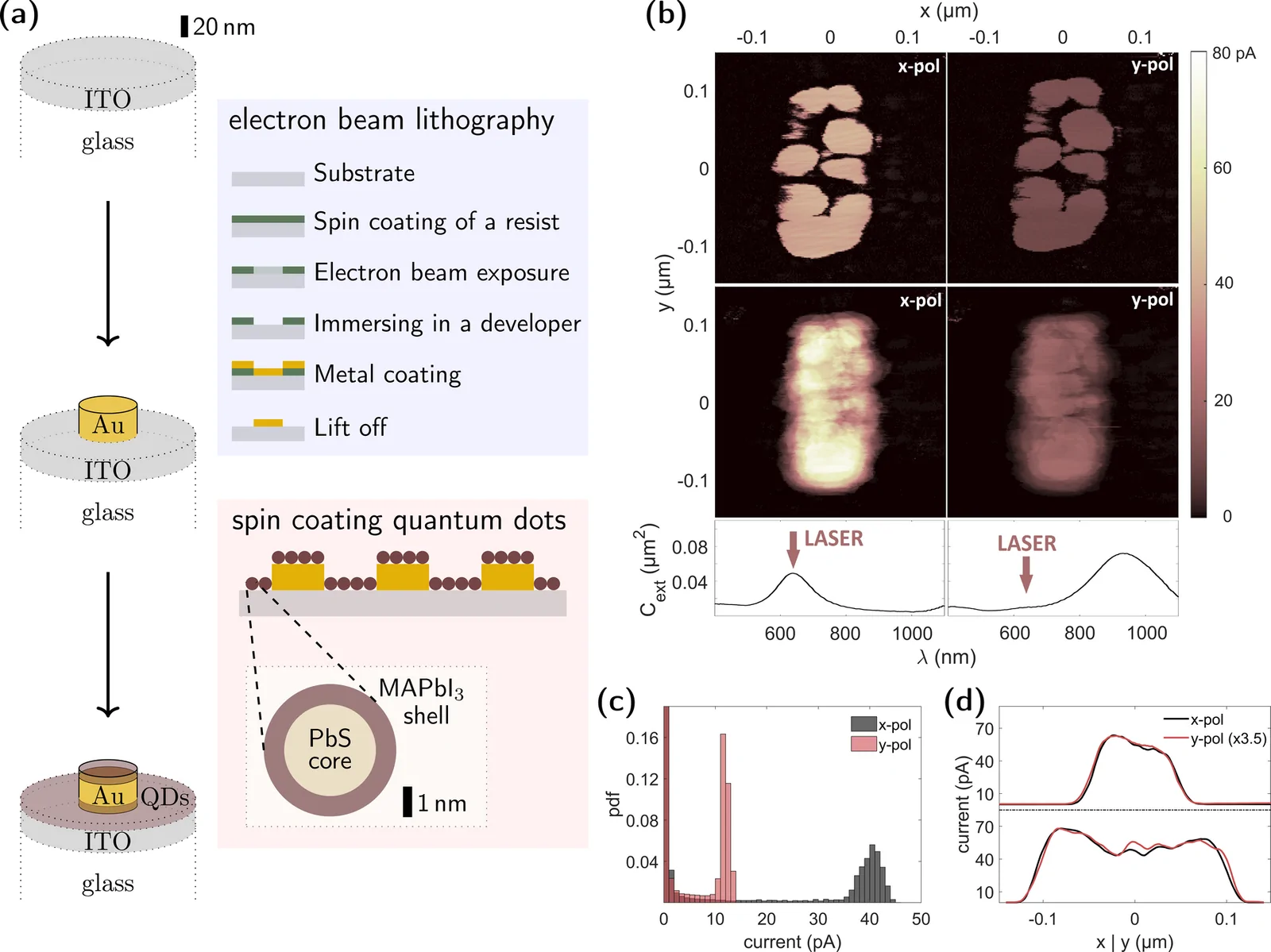
Sample preparation and pcAFM mapping. (a) Sketch of the sample preparation: electron beam lithography exposure, developing, metal coating and lift off is followed by the deposition of a compact PbS/MAPbI_3_ QD monolayer by spin coating. (b) pcAFM maps of QD-covered rectangular gold cuboids excited in the focus of a 1.45 N.A. objective (wavelength 633 nm, laser power 0.06 *μ*W). Single particle pcAFM maps for resonant (*x*-polarization, short axis) and off-resonant (*y*-polarization, long axis) excitations are shown in the top row. Averaged maps of 12 nominally identical particles are shown in the middle row. The bottom row depicts the corresponding extinction spectra of nominally identical particles in a 300 nm square array. (c) Current distribution histograms of the single particle maps. (d) Current cross sections along the short (top panel) and the long axis (bottom panel) of the averaged photocurrent maps. The current values for the off-resonant (*y*-polarized) sections are multiplied by a factor of 3.5.

In the photocurrent AFM (pcAFM), PtIr-metallized silicon tips (Bruker SCM-PIT-V2) were operated in contact mode in the force range of 5-20 nN. The photocurrents were detected with an *I*/*V* amplifier (1 nA/V, root-mean-square noise level 55 fA), with the tip grounded and the bias applied to the sample. The beam of either a stabilized helium neon laser (wavelength 633 nm) or a tunable supercontinuum source was expanded to a diameter of 10 mm and focused through an immersion microscope objective with a numerical aperture (N.A.) of 1.45. With the focus position fixed, the tip was scanned.

The pcAFM scans at zero bias on QD-covered gold rectangular cuboid nanoparticles, excited at a wavelength of 633 nm show areas of uniform current that are separated by areas of zero current, see Figure 1(b) and the current histograms in Figure 1(c). We note that on bare gold particles (i.e., without QD coverage) we observe similar spatially selective contact, however with much higher currents driving the amplifier into saturation (at 10 nA). The same pattern is observed on a given particle for polarization-dependent resonant (along the short particle axis) and nonresonant (along the long particle axis) excitation, shown on the left and right hand side of the top row in Figure 1(b), respectively. The corresponding extinction spectra (of an array of nanoparticles with identical dimensions) are depicted in the bottom row. By averaging the measured current patterns over 12 nominally identical particles, the current structure on the individual cuboids is smoothed out (second row in Figure 1(b)). The extent of the observed zero-current areas on the particles depends on the particle size and the normal force applied to the AFM tip (as demonstrated in the Supporting Information, Section S1), it is therefore most likely due to intermittent tip-sample contact. We find no indications of force-related sample degradation, in agreement with former work.^17^

We observe a ratio of 3.5 in the photocurrents for resonant and nonresonant excitations, as evidenced by the cross-cuts in Figure 1(d). This value corresponds approximately to the ratio of the particle’s absorption cross sections for the two chosen polarization directions at the excitation wavelength of 633 nm (see Supporting Information, Section S2). Interestingly, we find no indication of a polarization-dependent dipolar plasmon pattern in the individual photocurrent maps. At first view, this contrasts the spatial resolution below 100 nm that we have recently demonstrated with a PtIr tip on the same QD monolayer system.^17^ However, in that case the QDs were deposited on an ITO layer, so that the photocurrent was exclusively due to excitons generated in the QDs. In contrast, the current measured here on the gold nanoparticles, as we show later, originates mainly from hot holes that are excited in the particles before being injected into the QD layer. We can thus attribute the observed uniform photocurrent over the whole particle surface to the efficient transport of injected holes in colloidal PbS QD films,^22–24^ in contrast to the less diffusive electron/hole pairs that are directly excited in the QDs.^25,26^ In the following, we exploit this averaging effect to our advantage, as the position-independent photocurrent mitigates the challenges of positional accuracy and drift and thus allows measuring time-consuming *I*/*V* curves and wavelength sweeps on single nanoparticles.

As sketched within the energy diagram of a tip-contacted QD-covered gold nanoparticle in Figure 2(a), three contributions can make up the current in our samples. *I*_*ph*_ corresponds to the carriers photoexcited in the QDs, while *I*_*th*_ is due to thermionic emission in case of an asymmetric barrier height for thermal carriers (in the case of an applied bias; see Supporting Information, Section S3). Hot charges generated via photon and/or plasmon absorption in the metal particle (electrode) give rise to *I*_*el*_.

**Figure 2:**
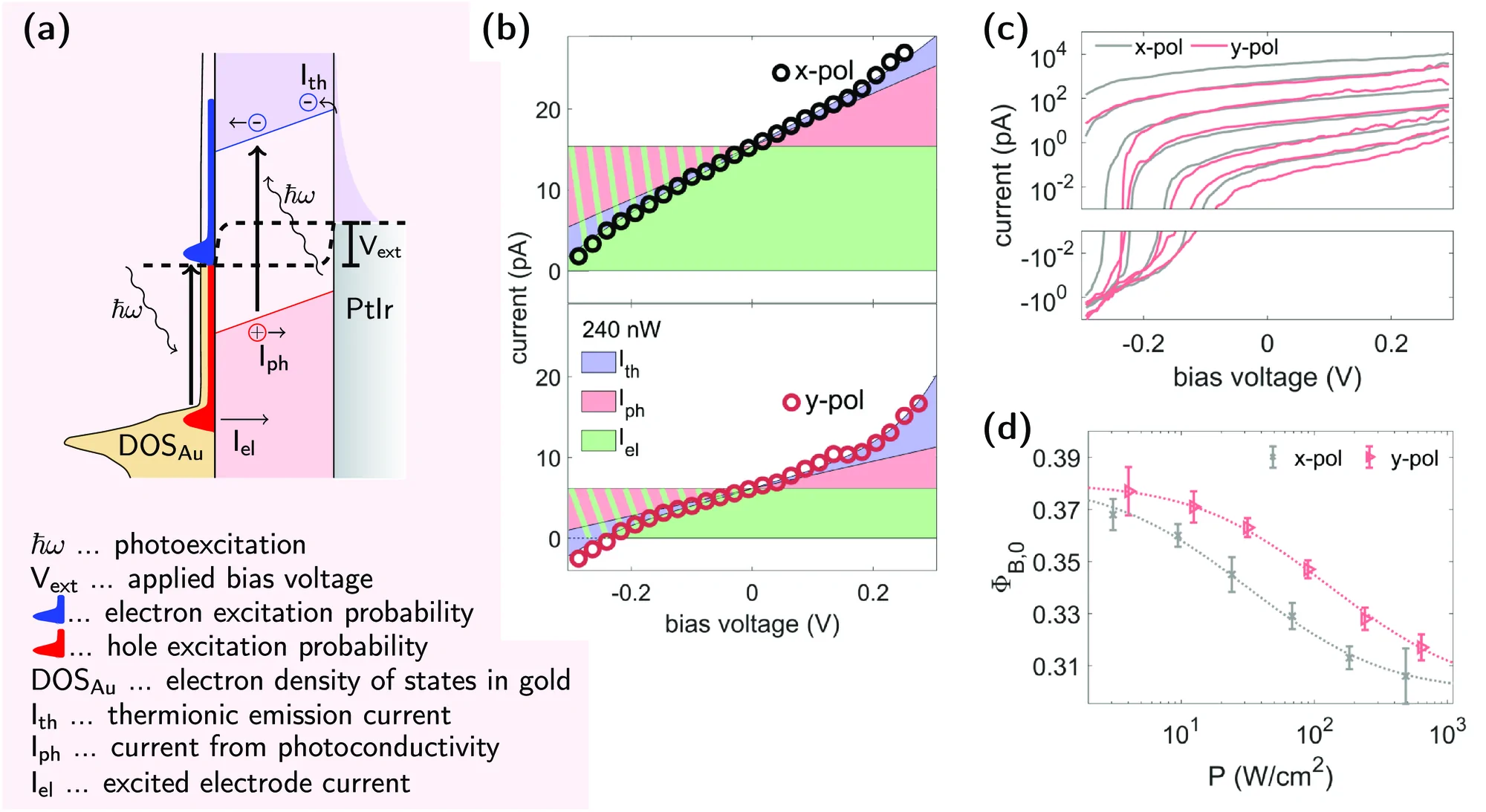
(a) Schematic of the energy levels and the three current contributions in a photoexcited ultrathin double Schottky barrier system with gold and PtIr as the electrodes. Asymmetric excitation probabilities with respect to the Fermi level for hot electrons and hot holes are expected due to the high d-band density of states in gold and the high efficiency of interband transitions. (b) *I*/*V* curves measured on a rectangular gold nanocuboid, for polarization directions parallel (x-polarization, black) and perpendicular (y-polarization, red) to the short particle axis and a laser power of 240 nW. The three current contributions *I*_*ph*_, *I*_*th*_ and *I*_*el*_ according to eqs 1–3 are indicated. In the hatched areas the bias-dependent current contributions have opposite sign with respect to the bias-independent hot hole current from the gold particle. (c) Full set of *I*/*V* curves measured with a laser power of 4 nW, 13 nW, 30 nW, 90 nW, 240 nW and 650 nW entering the microscope (at a wavelength of 633 nm), before being focused through a 1.45 N.A. objective. (d) Potential barriers *Φ*_B,0_ as extracted from the corresponding fit parameters. The dotted lines indicate the barrier heights derived from a two-level-model, as discussed in the Methods file of the Supporting Information.

From a previous pcAFM study,^16^ we know that the photocurrent *I*_ph_ from the PbS/MAPbI_3_ QDs is given by

1 $$I_{\mathrm{ph}}\propto P^{\alpha }\left(V_{\mathrm{ext}}+V_{\mathrm{bi}}\right)$$

following a power law with an exponent *α* of 0.64 with respect to the irradiance *P* and a linear relationship to the sum of the built-in voltage *V*_bi_ and the externally applied bias voltage *V*_ext_.

The thermionic emission current *I*_th_ can be estimated for the case of *V*_bi_ ≈ 0 by

2 $$I_{\mathrm{th}}=-sFA^{*}T^{2}\exp\left(-\frac{e\Phi_{B}}{k_{B}T}\right)\exp\left(-\frac{esV_{\mathrm{ext}}}{n_{s}k_{B}T}\right)\left(1-\exp\left(\frac{esV_{\mathrm{ext}}}{k_{B}T}\right)\right)$$

as described in detail in the Supporting Information (Section S3), with *s* ≔ sign­(*V*_ext_), the contact area *F*, the Richardson constant A*, the temperature *T*, the Boltzmann constant *k*_B_, the ideality factors *n*_*s*_, the elementary charge *e*, and the Schottky barrier heights *Φ*_B_, here assumed identical at the two electrode-semiconductor interfaces, since this model is intended to be applied to the electrode pair of gold on glass and a PtIr coated AFM-tip, for which similar work functions are reported^27,28^ and thus similar Schottky barrier heights are expected.

Figure 2(b) shows two exemplary *I*/*V* curves, measured on rectangular gold nanocuboids for polarization directions along the short (*x*-polarization) and the long axis (*y*-polarization). The nonlinearity in the bias range of ±0.25 V and the similar work functions of gold and PtIr (*V*_bi_ ≈ 0, see the Methods file of the Supporting Information), allows the data to be fitted with the thermionic emission model (eq 2), supplemented by a linear term. By assigning the voltage-dependent part of the linear contribution to *I*_ph_ and the offset at *V*_ext_ = 0 to *I*_el_, the indicated bias-dependent weights of the three current contributions are obtained.

Figure 2(c) shows a full set of measured *I*/*V* curves under varying laser power. The extinction spectra corresponding to the two polarization directions as depicted in the bottom row of Figure 1(b) highlight that the laser wavelength of 633 nm is resonant for *x*-polarized and off-resonant for *y*-polarized excitation. From the thermionic emission contribution obtained by the fitting routine (Figure 2(b)) we deduce an irradiance-dependent Schottky barrier height *Φ*_B_, plotted in Figure 2(d) for an assumed contact area of 30 nm^2^ (ref 17). The irradiance dependence, in turn, can be fitted by a two-state-model with a time constant of 8 ps for both, *x*- and *y*-polarization. For details on this two-state-model see the Methods file of the Supporting Information.

The current due to hot carriers generated by photon and/or plasmon absorption in the nanoparticles *I*_el_ is expected to follow the absorption rate of the corresponding quanta, i.e., to depend linearly on the irradiance. The bias dependence of *I*_el_ is expected to reflect the energy distribution of the hot carriers and thus the electrode material and the photon energy. In Figure 3(a) the hot carrier excitation probabilities for an excitation wavelength of 633 nm are sketched schematically for gold and aluminum, together with their energy levels in relation to the QD bandgap. For a detailed description of the PbS QD energy levels, see the Supporting Information, Section S4. The work function of PtIr is similar to gold and about 0.8 eV higher than aluminum (with potential deviations due to the presence of the glass substrate).^27–29^

**Figure 3:**
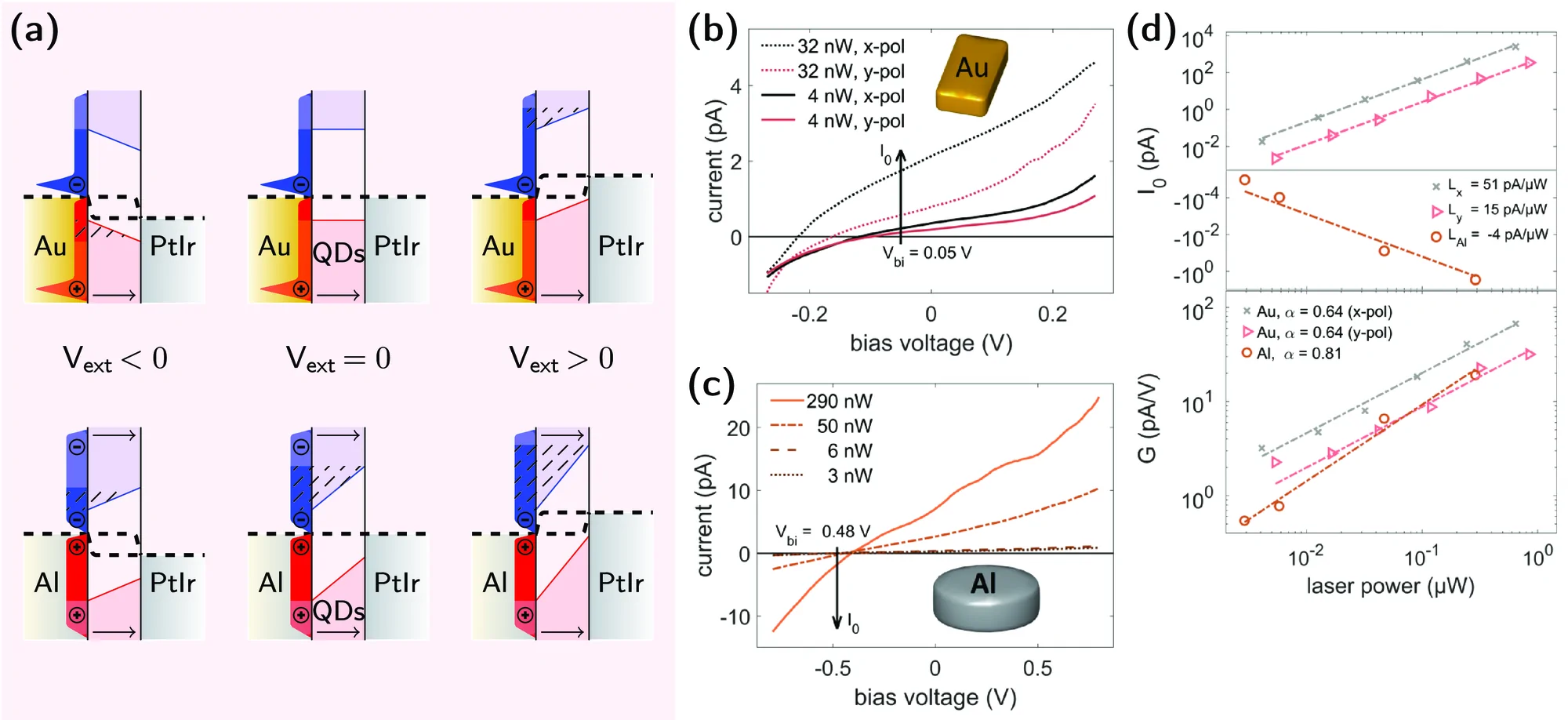
Gold and aluminum electronic structure and currents. (a) Sketches of the excitation probability densities of hot electrons (blue) and hot holes (red) in gold and aluminum nanoparticles separated from PtIr by QDs forming an ultrathin double Schottky barrier. The energy levels between the electrodes are drawn as expected for the used PbS/MAPbI_3_ QDs. Strong red and blue color indicate hot charges bound to the metal, whereas pale color indicate hot charges that can pass through the QD layer. The hatched areas indicate hot charges at an energy level that allows them to transfer to the QDs, but that is too low to overcome the electric field in the QDs. In the left row the case of a negative applied external voltage (*V*_ext_) is depicted. The resulting nonthermal equilibrium in the QDs is indicated by the splitting of the quasi-Fermi-levels of electrons and holes. The case of zero and positive applied external voltage are shown in the middle and the right rows, respectively. (b) *I*/*V* curves measured on a gold nanoparticle (from the set in Figure 2(c)), plotted on a linear scale. (c) *I*/*V* curves measured on an aluminum nanoparticle. The 633 nm wavelength laser excitation is resonant for the aluminum disk-shaped particle and for the rectangular gold cuboid when polarized parallel to the short side. (d) Double-logarithmic plot of the current under compensated built-in voltage conditions (*I*_0_) and of the conductance (*G*), as extracted from the linear part of the measured *I*/*V* curves. For *I*_0_ we find a linear relationship with respect to the applied laser power with the proportionality constants *L* given in the legend. For *G* we find a power law behavior with respect to the applied laser power with the exponents *α* as given in the legend.

In aluminum, the probability of exciting hot carriers is evenly distributed over the entire accessible energy range. We thus expect the current due to hot charges to depend rather linearly on the applied bias, with an electron-dominated current offset due to the low work function of aluminum. In contrast, gold has a peaked distribution due to the high density of states of the d-band and the efficient interband transitions.^30–32^ Due to the hole peak at energies exceeding the QD bandgap and the electron peak within the QD bandgap we expect a hole-dominated current from the gold particles that is only weakly dependent on small bias variations.

These expectations are confirmed by the experimental *I*/*V* curves on gold and aluminum nanoparticles, as plotted in Figs. 3(b) and (c), respectively. For a closer look we analyze the current offset *I*_0_ at *V*_ext_ + *V*_bi_ = 0, i.e., under compensated built-in voltage conditions. In this case, the electric field in the QD layer vanishes and thus charges excited in the QDs do not contribute to the current. The built-in voltages are derived by extrapolating the determined open circuit voltage to zero laser power by exploiting the fact that the current of hot carriers from the metal depends linearly on the laser power, while the photoconductivity of the QD layer decreases sub-linearly towards zero.^16^ A detailed description of the procedure is given in the Methods file of the Supporting Information. This effect is also demonstrated by the current maps of a high N.A. focus on a flat gold surface, as depicted in the Supporting Information. After showing that the measured current maps reflect the simulated focus patterns with high accuracy (Section S5), a bias variation demonstrates the intensity-dependent weighting of the current contributions (Section S6). In brief, in the low-intensity part of the focus pattern, moderate negative bias values are observed to generate negative currents. In contrast, in the high-intensity parts, positive current values prevail for increasingly negative biases (only to flip sign at some negative bias threshold).

In the case of the gold/PtIr combination, the built-in voltage is close to zero due to the similar work functions.^27–29^ Therefore, also the thermionic emission at compensated built-in voltage is negligible, which allows us to attribute *I*_0_ entirely to charges excited in the gold. For the Al/PtIr pairing we observe a built-in voltage of about 0.5 V. Since the *I*/*V* curves are almost linear in the ±0.7 V bias range we can conclude that also in this case the thermionic emission contribution is negligible under compensated built-in voltage conditions.

Consistent with the energy landscape sketch in Figure 3(a) we find for the gold particle a positive linear correlation between *I*_0_ and the laser power. This corresponds to positive charge, i.e. hole transport. For the aluminum particle with its lower work function we observe a negative linear correlation between *I*_0_ and the laser power, corresponding to negative charge, i.e. electron transport. The extracted values for *I*_0_ from the *I*/*V* curves are given in the top plot of Figure 3(d), together with a linear fit for the laser power dependence. Due to the increasing laser power step sizes chosen for the measurement, the data are presented in a double-logarithmic scale (making arbitrary power law relations appear as a straight line). The assumed linear dependence of the hot charge current on the laser power is evidenced by the excellent agreement of the data points with the linear fit function.

The slopes of the *I*/*V* curves in the linear region around zero bias, corresponding to the conductance *G*, are summarized in the bottom plot of Figure 3(d). For both, gold and aluminum we derive an approximate power law dependence on the laser power. The 0.64 exponent found for gold is the very value measured on weakly absorbing ITO electrodes in previous studies.^16,17,21^ This indicates that the bias-dependent part of the hot carrier current with its expected linear power dependence is negligible, compared to the photocurrent from the QDs. With a view on Figure 3(a), it has to be concluded that the excitation probability for holes in the hatched area is significantly lower than for d-band holes. On the other hand, on the aluminum disk we find an exponent of 0.81. This hints towards an approximate power law dependence due to the superposition of power laws with exponents of 0.64 and 1, indicating that the flat hot carrier distribution in aluminum gives rise to a linear bias dependence of the hot charge current that comes in a similar proportion as the photocurrent from the QDs.

The anisotropic geometry of the gold particles with their polarization-dependent absorption and scattering cross sections is also reflected in the current measurements, as plotted in Figure 3(d). The offset in *I*_0_ between the excitation with resonant and off-resonant polarization confirms that increased absorption and the related near-field amplification gives rise to an increase in the hot hole current from the particle. In addition, there is also an offset in the determined conductance G that lets us conclude that the conductivity in the QD layer is also effected by the enhanced near-field at resonant polarization. The offset regarding *I*_0_ is given by a multiplicative constant of 3.4, approximately corresponding to the expected difference in absorption cross-section of 4.4 (see Supporting Information, Section S2). Further, the offset in *G* is given by a multiplicative constant of 2.3 which is in good agreement with the absorption value difference, when taking into account the exponent of 0.64 for the power-law dependence of the QD photoconductivity (as an electric field intensity amplification of 4.4 corresponds to a conductance amplification of 2.6). This scaling with the absorption cross-section means that for the present case no fundamental difference in the energy distribution of hot charges originating from either direct photon absorption (off-resonant excitation) and plasmon absorption (resonant excitation) is observed. In particular, we find no evidence for a preferential contribution due to excitations from states near the Fermi level by the plasmon, in accordance with theoretical predictions for 40 nm thick gold films,^31^ but in contrast to gold films and particles in the size range below 10 nm, as well as for silver nanoparticles, where (geometry assisted) excitations from the Fermi level are expected.^10,33–35^

Based on the observations made so far, for the gold particle the hole current can be attributed to absorption, while the conductance is related to the associated field enhancement. In contrast, for the aluminum particle the hot carrier current due to absorption shows a bias dependence and thus also contributes to the conductance of the system. We can summarize the current *I*_el_ due to hot charges generated by plasmon decay in aluminum and gold to be approximated by

3 $$I_{\mathrm{el}}\propto \{\begin{array}{cc} C_{\mathrm{abs}}P\left(\Phi_{B,e}-\Phi_{B,h}+V_{\mathrm{ext}}+V_{\mathrm{bi}}\right), & \mathrm{for}\;\mathrm{Al}\\ C_{\mathrm{abs}}P, & \mathrm{for}\;\mathrm{Au} \end{array}$$

when assuming equal hole and electron transport efficiencies in the case of aluminum, with *C*_abs_ the absorption cross section of the respective metal particles and *Φ*_B,e_ and *Φ*_B,h_ the Schottky barrier heights for electrons and holes, respectively.

We now further exploit the current homogeneity over the whole particle surface by varying the laser wavelengths while scanning single square gold nanocuboids, aiming at a measurement of the spectral d-band excitation probability. We set the bias to zero, thus expecting to detect only hot charges from the gold, since *I*_th_ = 0 and *I*_ph_ ≈ 0 due to *V*_bi_ ≈ 0 for the gold/PtIr combination. Figure 4(a) shows the current maps measured on four individual square gold nanocuboids, where during scanning the illuminating wavelength has been changed stepwise from 440 to 655 nm, for a constant laser power of 0.5 *μ*W. The averaged current values as a function of excitation energy are plotted by the symbols in Figure 4(b). The averaged dataset contains only pixels with current values exceeding a threshold of 1 pA to exclude data points with intermittent tip-sample contacts as present in the central part of the particles.

**Figure 4:**
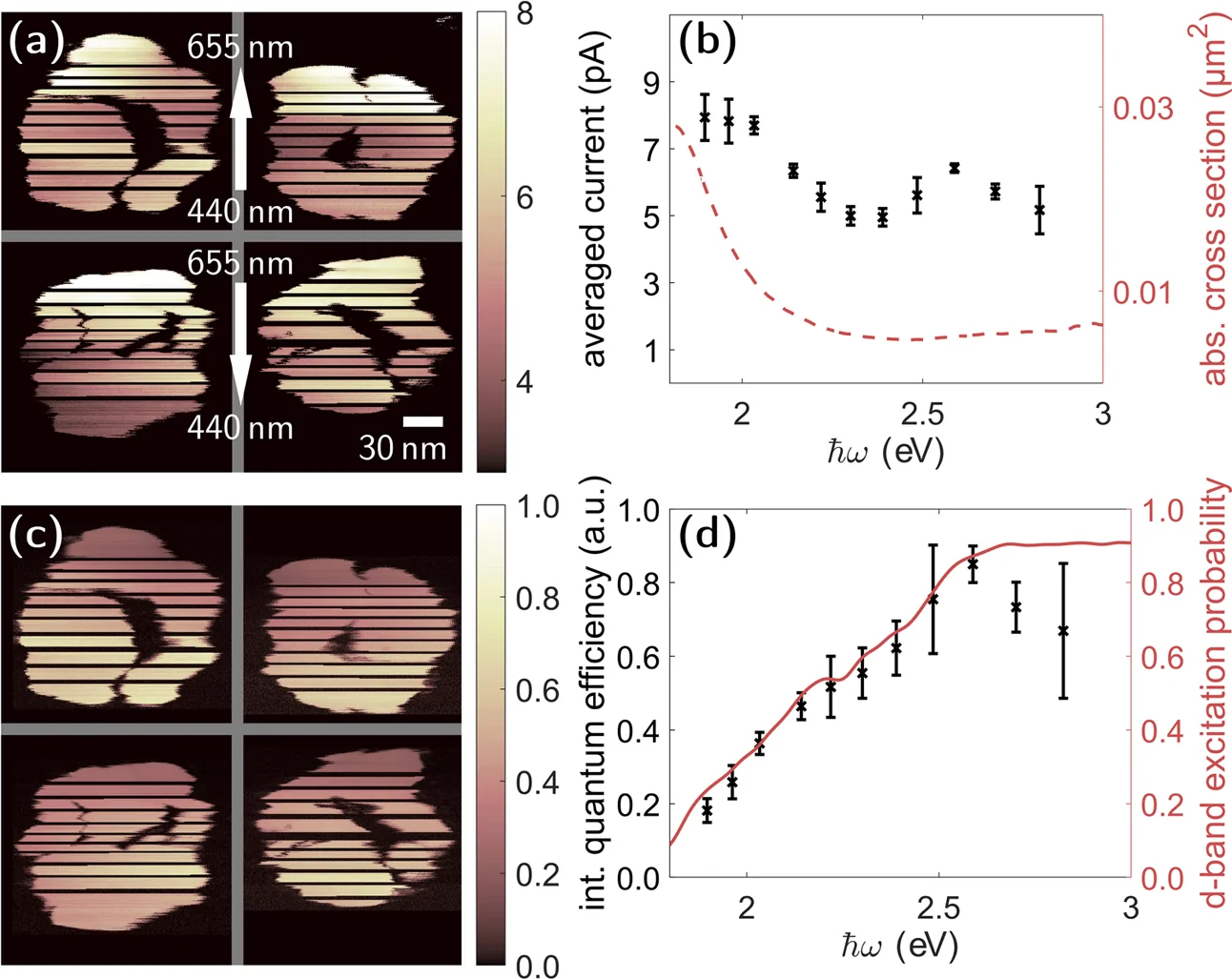
Wavelength-dependent maps and d-band spectroscopy. (a) Tip scan pcAFM maps of four different square gold nanocuboids. To ensure stable excitation conditions the particles are illuminated with a defocused 1.45 N.A. objective. The direction of the stepwise wavelength variation from 440 to 655 nm (2.8 eV to 1.9 eV) is indicated by the arrows, the laser power was held constant at 0.5 *μ*W throughout the scans. (b) Averaged current for each wavelength extracted from (a) (symbols), the error bars indicate the standard deviations of the four averaged measurements. The dashed line shows the estimated absorption cross section of the particles, deduced from the measured extinction cross section of an array of nominally identical particles. (c) Internal quantum efficiency maps, converted from (a) with the data in (b). (d) Averaged internal quantum efficiency (symbols), compared to the probability of exciting a hole in the d-band (line).

For the used particle geometry we estimate the absorption cross section from the extinction spectrum (measured on a particle array), based on a model described in the Supporting Information (section S2). The resulting absorption cross section is depicted by the dashed line in Figure 4(b). Next, we convert the current maps to maps of internal quantum efficiency, as depicted in Figure 4(c). Finally, we summarize the averaged internal quantum efficiencies in Figure 4(d) (symbols) and compare these data to the calculated d-band excitation probability (line), derived for plasmon decay in a 40 nm gold sphere by Brown et al.^30^ Excellent agreement is observed for all but the lowest wavelengths, i.e., the highest energy values. This can be understood as we consider hot hole transport only, which is no longer the case for photon energies above 2.6 eV. Here, d-band excitations can result in hot electrons with energies exceeding the QD band gap, as the d-band onset is 1.9 eV below the Fermi level and the QD band gap exceeds the Fermi level by about 0.7 eV.

This lets us conclude that the energy barrier provided by the QD monolayer allows for quantitative d-band hole spectroscopy. The key lies in the QD layer that is thin enough to ensure efficient carrier transport due to the light-induced occupation gradient between the electrodes, while being thick enough to prevent tunneling of excited carriers with energies within the bandgap.

In summary, scanning probe photocurrent measurements through an ultrathin Schottky barrier defined by a QD monolayer on plasmonic gold nanoparticles enable quantitative hot carrier spectroscopy. The accessible carrier type and energy range depend on the chosen QD type, making this measurement scheme a generally applicable spectroscopic analytical tool. Here, we have applied PbS/MAPbI_3_ with a core diameter of 3.2 nm to measure the excitation quantum efficiency of hot holes excited in gold in the 1.9–2.6 eV energy range. The hot carrier current from the metal is identified and separated from excitations in the QDs and thermionic emission by systematic variations of bias and laser power. Furthermore, by comparing gold with plasmonic aluminum nanoparticles, the role of the different electronic structure of the two metals is highlighted. We note that photothermal effects can be ruled out, since they would give rise to currents following the course of the absorption cross section, which is clearly not the case for the data in Figure 4(b). As photocurrent AFM combines these spectral capabilities with high spatial resolution, it could open significant new options for fundamental research, as well as for the characterization of novel materials relevant for photocatalysis, photodetection and solar energy conversion.

## Supplementary Material





## Notes

Methods: sample fabrication, two-state model estimation for the barrier height, built-in voltage determination (PDF)
Additional photocurrent AFM maps on gold nanoparticles with varying contact force and on a gold thin film with a high N.A. focus and bias variation, estimation of the absorption of gold particles, energy level and thermionic emission model for a thin double Schottky barrier, energy level scheme of the used PbS/MAPbI_3_ quantum dots (PDF)
