# Supplementary material for: Quantum-Dot-Based Scanning Probe d‑Band Hole Spectroscopy of Plasmonic Nanoparticles
Source: Nano Lett. 2026 Oct 1;26(40):14040–6. doi: 10.1021/acs.nanolett.6c03990 (PMC13674339; doi:10.1021/acs.nanolett.6c03990)
Supplement: Supplementary file 1 [file nl6c03990_si_001.pdf]

Methods:
Quantum-dot-based scanning probe d-band hole spectroscopy of plasmonic nanoparticles

Florian Küstner,[†] Harald Ditlbacher,[†] Andreas Hohenau,[†] Dmitry N. Dirin,[‡,¶] Maksym Kovalenko,[‡,¶] and Joachim R. Krenn[*,†]

†*Institute of Physics, University of Graz, 8010 Graz, Austria*

‡*Institute of Inorganic Chemistry, Department of Chemistry and Applied Biosciences, ETH Zürich, 8093 Zürich, Switzerland*

¶*Empa – Swiss Federal Laboratories for Materials Science and Technology, Laboratory for Thin Films and Photovoltaics, 8600 Dübendorf, Switzerland*

E-mail: joachim.krenn@uni-graz.at

M1 Sample fabrication

Glass coverslips 25 mm in diameter and 0.17 mm thick are covered by a 20 nm thick layer of ITO by electron beam evaporation, followed by 300°C annealing on a hot plate and ambient air plasma cleaning. By spin coating a solution with 2% poly(methyl methacrylate) at 3000 rpm we add a 100 nm thick resist film that is exposed in an electron beam lithography system. After chemical developing, 0.5 nm of chromium followed by 30 nm of gold, respectively 20 nm of aluminum are deposited on the substrate by physical vapor deposition. After liftoff in acetone at 45°C the QDs are spin coated with a solution with 10 g/L of PbS/MAPbI$_3$ (3.2 nm core diameter, 1 nm shell thickness, synthesized as reported before [1]) in 2,6-difluoropyridine at 2000 rpm.

M2 Two-state model estimation for the barrier height

To describe the observed irradiance dependence of the potential barrier Φ_{B}, we assume that after the absorption of a photon the gold/QD system is in any kind of exited state for the time τ (associated with a potential barrier $\Phi_{\mathrm{B}}^{\mathrm{e}}$) and then falls back to its ground state (associated with a potential barrier $\Phi_{\mathrm{B}}^{\mathrm{g}}$). The excitation rate $f_{g\to e}$ from the ground state to the excited state is given by the excitation rate f times the probability of finding the system in the ground state p_g. In steady state this excitation rate needs to be equal to the transition rate from the excited state to the ground state, $f_{e\to g}$, that is given by the probability of finding the system in the excited state p_e divided by its lifetime. This gives the condition

$$f_{g\to e} = f_{e\to g} \iff p_g f = \frac{p_e}{\tau} \iff (1-p_e)f = \frac{p_e}{\tau} \iff p_e = \frac{1}{1+\frac{1}{\tau f}}\,. \tag{M1}$$

The measured potential barrier Φ_B is given by

$$\begin{aligned}\exp\left(-\frac{e\Phi_\mathrm{B}}{k_\mathrm{B}T}\right) &= p_e \exp\left(-\frac{e\Phi_\mathrm{B}^\mathrm{e}}{k_\mathrm{B}T}\right) + p_g \exp\left(-\frac{e\Phi_\mathrm{B}^\mathrm{g}}{k_\mathrm{B}T}\right)\\ &= p_e\left(\exp\left(-\frac{e\Phi_\mathrm{B}^\mathrm{e}}{k_\mathrm{B}T}\right) - \exp\left(-\frac{e\Phi_\mathrm{B}^\mathrm{g}}{k_\mathrm{B}T}\right)\right) + \exp\left(-\frac{e\Phi_\mathrm{B}^\mathrm{g}}{k_\mathrm{B}T}\right),\end{aligned}$$

with the elementary charge e, the Boltzmann constant k_B and the temperature T. Substituting the excitation rate by $f = C^*_\mathrm{abs}P$ (with $C^*_\mathrm{abs} = \frac{C_\mathrm{abs}}{\hbar\omega}$ the absorption cross section in terms of absorbed photons, P is the incident irradiance), we get

$$\frac{1}{\exp\left(-\frac{e\Phi_\mathrm{B}}{k_\mathrm{B}T}\right) - \exp\left(-\frac{e\Phi_\mathrm{B}^\mathrm{g}}{k_\mathrm{B}T}\right)} = \frac{1 + \frac{1}{\tau C^*_\mathrm{abs}}\frac{1}{P}}{\exp\left(-\frac{e\Phi_\mathrm{B}^\mathrm{e}}{k_\mathrm{B}T}\right) - \exp\left(-\frac{e\Phi_\mathrm{B}^\mathrm{g}}{k_\mathrm{B}T}\right)}. \tag{M2}$$

We thus expect a linear relation between $\left(\exp\left(-\frac{e\Phi_\mathrm{B}}{k_\mathrm{B}T}\right) - \exp\left(-\frac{e\Phi_\mathrm{B}^\mathrm{g}}{k_\mathrm{B}T}\right)\right)^{-1}$ and P^{-1}. The fitted model as plotted by the dashed lines in Fig. 2(c) in the main text is in excellent agreement with the experimentally derived data and gives identical barrier heights of $\Phi_\mathrm{B}^\mathrm{g} = 0.38\,\mathrm{V}$ and $\Phi_\mathrm{B}^\mathrm{e} = 0.30\,\mathrm{V}$ for both polarization directions. From extinction spectroscopy data, as described in detail in the Supporting Information (section S2) we get $4.4\,C^*_\mathrm{abs,y} \sim C^*_\mathrm{abs,x} = 9 \cdot 10^8\,\frac{\mathrm{cm}^2}{\mathrm{J}}$, yielding from the model parameters a state lifetime of $\tau = 8\,\mathrm{ps}$ for both x- and y-polarized excitation. This lifetime corresponds approximately to the expected hot charge relaxation time due to electron-phonon coupling[2] and also to the time constants found in transient absorption measurements of the PbS/$MAPbI_3$ QD films.[3]

M3 Built-in voltage determination

The photocurrent of a PbS/$MAPbI_3$ QD monolayer can be described by

$$I_{ph} = C_{\mathrm{ph}} P^{0.64} \left(V_{\mathrm{ext}} + V_{\mathrm{bi}} \right) , \tag{M3}$$

with a proportionality constant C_{ph}, the applied bias V_{ext} and the built-in voltage V_{bi}.[1] Further, the injection current I_{el} of hot charges from the electrodes is expected to depend linearly on the applied irradiance. When an asymmetric hot charge distribution in the metal particles with respect to the QD bandgap exists (as for gold and aluminum, see Fig. 3(a) in the main text), then the injection current can be approximated by

$$I_{el} = C_{\mathrm{el}} P, \tag{M4}$$

with a non-zero injection constant C_{el}. In the case of negligible thermionic emission (linear I/V characteristic), the total current I can be approximated by

$$I = C_{\mathrm{ph}} P^{0.64} \left(V_{\mathrm{ext}} + V_{\mathrm{bi}} \right) + C_{\mathrm{el}} P \, . \tag{M5}$$

For the external bias V_0 that gives zero current we therefore obtain with

$$V_0 = -\frac{C_{\mathrm{el}}}{C_{\mathrm{ph}}} P^{0.36} - V_{\mathrm{bi}} \, , \tag{M6}$$

a relationship that allows for the determination of V_{bi} from the knowledge of $(V_0, P^{0.36})$ pairs, which we obtain from fitting the linear part of the I/V curves in Fig. 2(b) in the main text for gold and in Fig. 3(c) in the main text for aluminum. The corresponding values are plotted in Fig. M1, demonstrating the linear relationship between V_0 and $P^{0.36}$. The extracted values for the built-in voltage are $0.08 \pm 0.05\,\mathrm{V}$ and $0.03 \pm 0.05\,\mathrm{V}$ for x- and y-polarization on gold, respectively, and $0.48 \pm 0.01\,\mathrm{V}$ for aluminum.

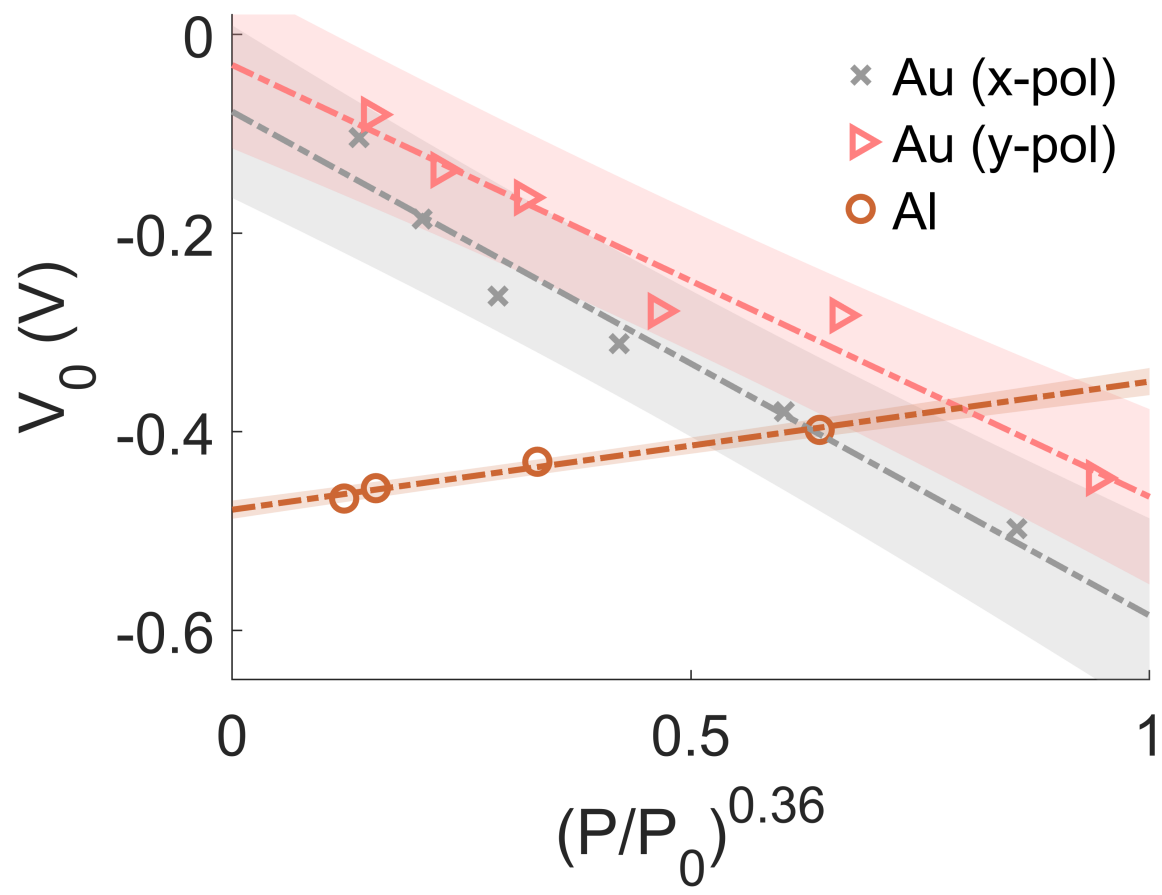

Figure M1: Measured laser power (P) dependence of the zero-crossing voltage V_0 for a rectangular gold nanocuboid (both polarization directions) and for an aluminum nanodisk (symbols). The scaling factor P_0 is chosen as $1\,\mu$W. The lines represent the fitted linear models, with the shaded areas indicating the 90% confidence intervals.

References

(1) Küstner, F.; Ditlbacher, H.; Hohenau, A.; Dirin, D.; Kovalenko, M.; Krenn, J. Quantitative photocurrent scanning probe microscopy on PbS quantum dot monolayers. *Nanoscale* **2024**, *16*, 16664–16670.

(2) Ahmadi, T. S.; Logunov, a. S. L.; El-Sayed, M. A. Picosecond dynamics of colloidal gold nanoparticles. *J. Phys. Chem.* **1996**, *100*, 8053–8056.

(3) Grimaldi, D. An investigation of light-induced charge carrier transport and ultrafast dynamics at the nanoscale. Ph.D. thesis, University of Graz, 2023.
