# Supplementary material for: Quantum-Dot-Based Scanning Probe d‑Band Hole Spectroscopy of Plasmonic Nanoparticles
Source: Nano Lett. 2026 Oct 1;26(40):14040–6. doi: 10.1021/acs.nanolett.6c03990 (PMC13674339; doi:10.1021/acs.nanolett.6c03990)
Supplement: Supplementary file 2 [file nl6c03990_si_002.pdf]

Supporting Information: Quantum-dot-based scanning probe d-band hole spectroscopy of plasmonic nanoparticles

Florian Küstner,[†] Harald Ditlbacher,[†] Andreas Hohenau,[†] Dmitry N. Dirin,[‡,¶] Maksym Kovalenko,[‡,¶] and Joachim R. Krenn[*,†]

†*Institute of Physics, University of Graz, 8010 Graz, Austria*

‡*Institute of Inorganic Chemistry, Department of Chemistry and Applied Biosciences, ETH Zürich, 8093 Zürich, Switzerland*

¶*Empa – Swiss Federal Laboratories for Materials Science and Technology, Laboratory for Thin Films and Photovoltaics, 8600 Dübendorf, Switzerland*

E-mail: joachim.krenn@uni-graz.at

S1 Photocurrent AFM maps on gold nanoparticles with varying contact force

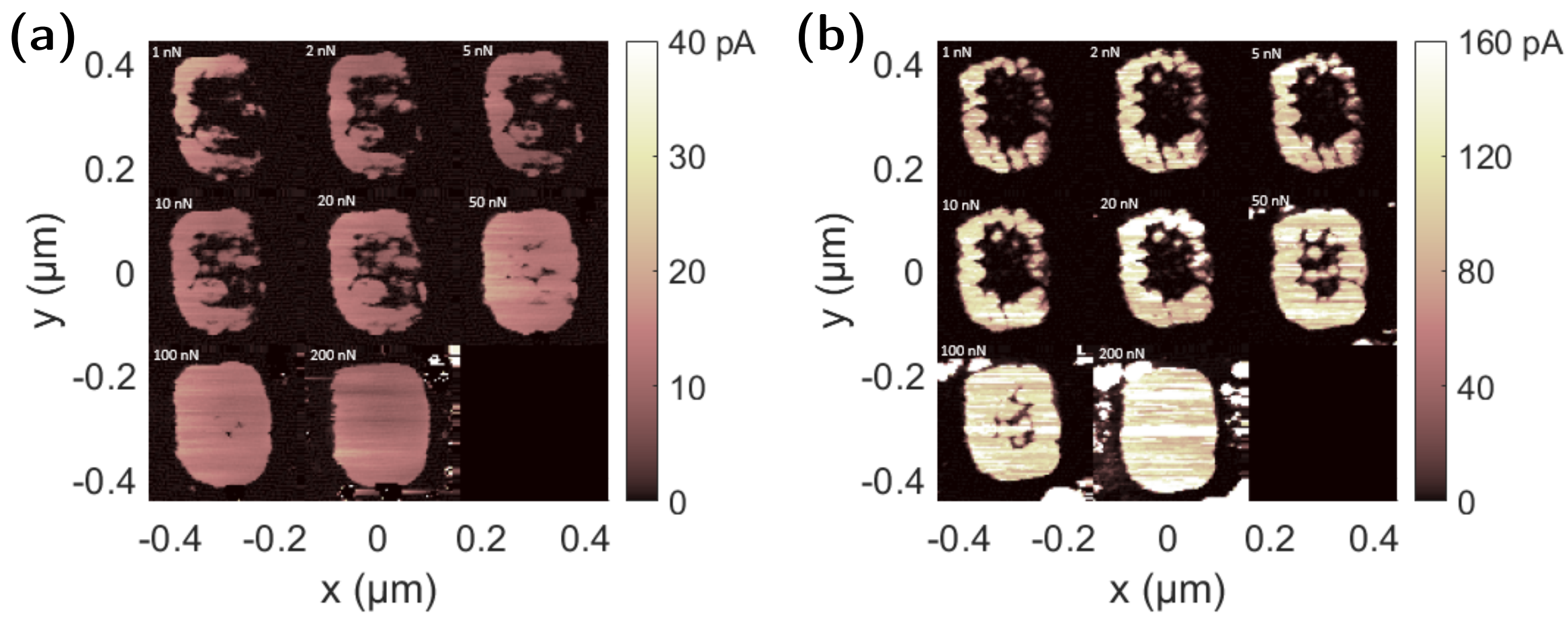

Figure S1: Photocurrent AFM contact force variation on rectangular gold nanocuboids covered by a PbS/$MAPbI_3$ QD monolayer. Here, the gold particles have a nominal size of $110 \times 140 \times 30\,nm^3$, the contact force is increased from 1 nN (top left) to 200 nN (bottom center) and the scan direction is from left to right. Panels (a) and (b) show measurements at 0.0 V (laser power 10 μW) and 0.8 V bias (laser power 4 μW), respectively. The laser (wavelength 730 nm, polarization parallel to the short particle side) is focused by a 0.3 N.A. objective, the power values were measured before the laser enters the objective. A larger contact force increases the ratio of contacted and uncontacted areas on top of the particles, the measured current under contact is however vastly independent of the contact force.

S2 Estimation of the absorption of gold particles

For ellipsoidal particles significantly smaller than the wavelength (quasistatic approximation) the analytic expression for the polarizability α for the simplest case of a sphere is given by[1]

$$\alpha_{\mathrm{S}} = 3V \frac{\epsilon(\omega) - \epsilon_{\mathrm{m}}}{\epsilon(\omega) + 2\epsilon_{\mathrm{m}}} \tag{S1}$$

with the particle volume V, permittivity of the surrounding medium ϵ_{m} and the the permittivity of the particle $\epsilon(\omega)$ depending on the frequency ω. The resonance frequency ω_{res} is than implicitly given by

$$\epsilon_1(\omega_{\mathrm{res}}) + 2\epsilon_{\mathrm{m}} = 0 \tag{S2}$$

with the real part of the permittivity of the particle $\epsilon_1(\omega)$. For other geometries or an inhomogeneous surrounding medium numerical methods are necessary to theoretically predict the properties of the localized surface plasmon resonances.[2] However, taking the QD layer into account is only possible using rough approximations, thus here a simplified description of the damping processes is derived on the basis of the polarizability of a sphere, the Drude permittivity of a metal and parameters fitted to experimentally obtained linewidths. Therefore, rectangular gold nanocuboids with lengths and widths varied between 30 nm and 150 nm in 10 nm steps and a height of 30 nm were fabricated by electron beam lithography, evenly arranged in square lattices with particle distances of 300 nm. To extract the spectral linewidth corresponding to the damping constant γ, the measured extinction spectra are fitted with the spectral line profile[2]

$$C_{\mathrm{ext}}(\omega) = 4\pi \frac{\epsilon_{\mathrm{m}}}{\epsilon_0 c} \frac{\gamma \omega_{\mathrm{res}}^3 \omega}{(\omega_{\mathrm{res}}^2 - \omega^2)^2 + \gamma^2 \omega_{\mathrm{res}}^2} V = \frac{K\omega}{(\omega_{\mathrm{res}}^2 - \omega^2)^2 + \gamma^2 \omega_{\mathrm{res}}^2}, \tag{S3}$$

of a small spherical metallic particle, with the vacuum permittivity ϵ_0, the speed of light c and an additional fit parameter K to approximate the inhomogeneous permittivity of the surrounding medium. In Figure S2 the full set of derived damping values $\Gamma = \hbar\gamma$ are plotted

with respect to the particle size parallel and perpendicular to the incident light polarization. The total damping of the plasmon resonance can be expressed by the sum[3]

$$\Gamma = \Gamma_{\mathrm{b}} + \Gamma_{\mathrm{IB}} + \Gamma_{\mathrm{rad}} + \Gamma_{\mathrm{surf}} \,, \tag{S4}$$

of a bulk damping Γ_{b}, interband damping Γ_{IB}, radiation damping Γ_{rad} and surface damping Γ_{surf} contribution. While Γ_{b} and Γ_{IB} are material inherent properties, Γ_{rad} and Γ_{surf} depend strongly on the particle size and shape. For small spherical particles the non-radiative damping contribution can be approximated by[1,4]

$$\Gamma_{\mathrm{non-rad}} = \left. \frac{2\hbar\epsilon_2}{\left|\frac{d\epsilon_1}{d\omega}\right|} \right|_{\omega=\omega_{\mathrm{res}}} \,, \tag{S5}$$

where ϵ_1 and ϵ_2 are the real and the imaginary part, respectively, of the dielectric permittivity. For photon energies below 1.76 eV the dielectric function $\epsilon(\omega)$ of gold is well described by the modified Drude model[5,6]

$$\epsilon(\omega) = \epsilon_\infty - \frac{\omega_{\mathrm{p}}^2}{\omega^2 + \mathrm{i}\omega\gamma} = \epsilon_\infty - \frac{\omega_{\mathrm{p}}^2}{\omega^2 + \gamma^2} + \mathrm{i}\frac{\omega_{\mathrm{p}}^2\gamma}{\omega\left(\omega^2 + \gamma^2\right)} \,, \tag{S6}$$

with the dielectric response of bound valence electrons ϵ_∞ (Ref.[2]) and the plasma frequency ω_{p}. By interpreting the dephasing γ as an inverse scattering time

$$\gamma = 2\pi\nu_{\mathrm{F}} \sum_j \frac{1}{l_j} = \gamma_{\mathrm{b}} + \frac{\nu_{\mathrm{F}} A}{l_{\mathrm{eff}}} \,, \tag{S7}$$

with the Fermi velocity ν_{F} and the mean free paths l_j for various scattering processes $j \in \{1, 2, ...\}$, also surface scattering in finitely sized particles can be included by combining the particle size independent bulk contributions into the bulk damping constant γ_{b} and describing the size effect by an effective particle length l_{eff} and the surface damping constant A.[7] In the range of $1.76\,\mathrm{eV} < \hbar\omega < 2.2\,\mathrm{eV}$, where interband damping is present but

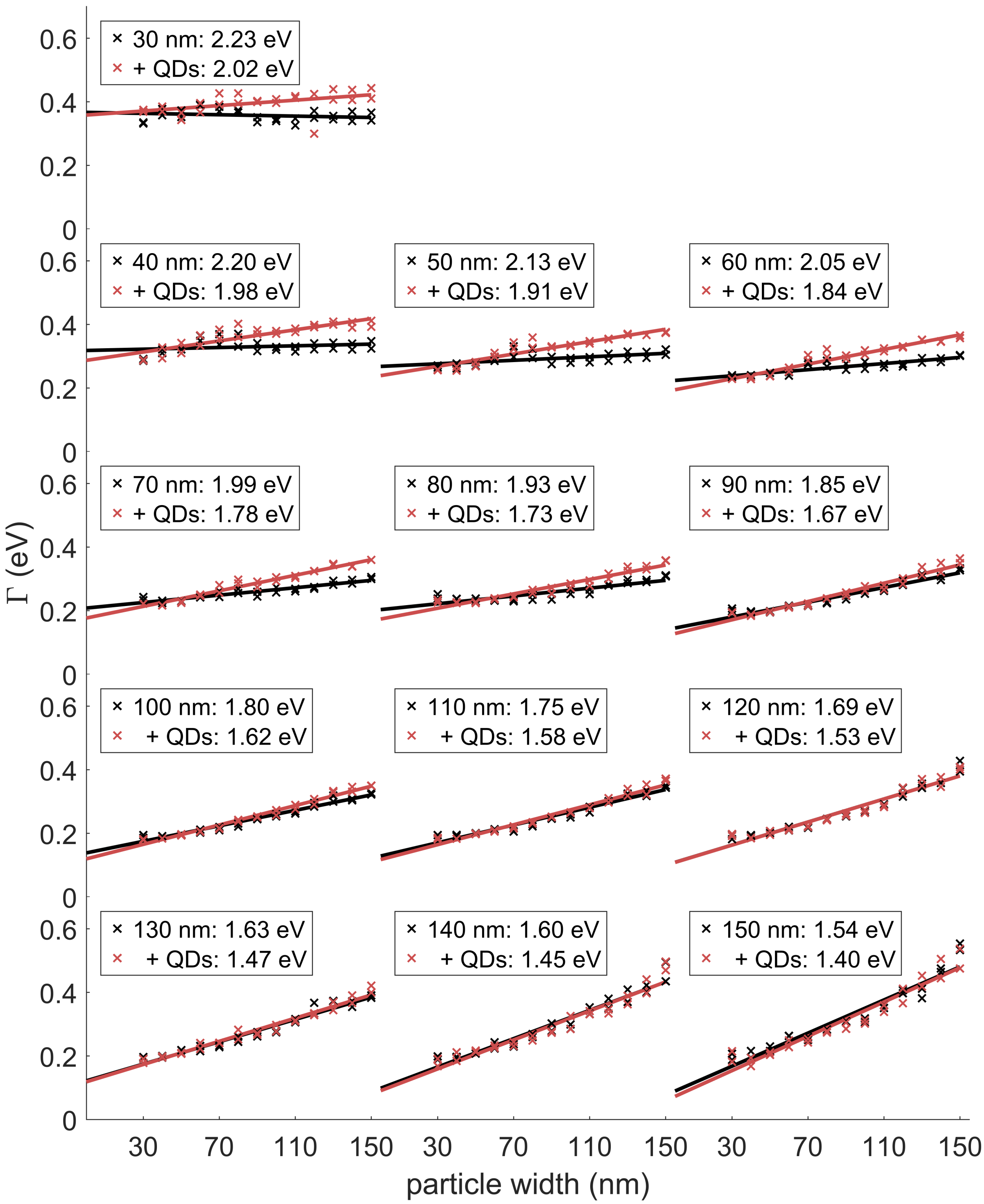

Figure S2: Full set of derived damping values grouped by particle length. In the legend of each subplot the associated particle length parallel to the polarization direction is given, together with the averaged resonance energy without and with QDs. The abscissa gives the particle widths perpendicular to the polarization direction. The full lines are linear fits for the bare (black) and the QD-covered gold particles (red).

weak, the dielectric function of gold can be approximated by adding the interband contribution $\epsilon_{2,\mathrm{IB}}$ of gold to the imaginary part of the Drude dielectric function[3] given in equation S6. In the visible and near infrared spectral range with $\gamma_\mathrm{b} = 0.11\,\mathrm{fs}^{-1} << \omega$ the dielectric permittivity of gold can thus be estimated by

$$\epsilon(\omega) = \epsilon_\infty - \frac{\omega_\mathrm{p}^2}{\omega^2} + \mathrm{i}\left(\frac{\omega_\mathrm{p}^2}{\omega^3}\left(\gamma_\mathrm{b} + \frac{\nu_\mathrm{F} A}{l_\mathrm{eff}}\right) + \epsilon_{2,\mathrm{IB}}(\omega)\right) . \tag{S8}$$

The derivative of the real part of the dielectric permittivity with respect to the frequency is thus given by

$$\left|\frac{d\epsilon_1}{d\omega}\right| = \frac{2\omega_\mathrm{p}^2}{\omega^3} , \tag{S9}$$

and the non-radiative contributions Γ_b, Γ_IB and Γ_surf can be assigned to[3]

$$\Gamma_\mathrm{b} = \hbar\gamma_\mathrm{b} = 72\,\mathrm{meV} , \tag{S10}$$

$$\Gamma_\mathrm{IB} = \epsilon_{2,\mathrm{IB}}(\omega_\mathrm{res})\frac{\hbar\omega_\mathrm{res}^3}{\omega_\mathrm{p}^2} , \tag{S11}$$

and

$$\Gamma_\mathrm{surf} = \frac{\hbar\nu_\mathrm{F} A}{l_\mathrm{eff}} . \tag{S12}$$

The radiative damping contribution can be obtained from the ratio of the scattering cross section C_sca to the absorption cross section C_abs (Ref. [1])

$$\Gamma_\mathrm{rad} = \Gamma_\mathrm{non-rad} \left.\frac{C_\mathrm{sca}(\omega)}{C_\mathrm{abs}(\omega)}\right|_{\omega=\omega_\mathrm{res}} , \tag{S13}$$

with

$$C_\mathrm{sca}(\omega) = \frac{k^4(\omega)}{6\pi}\left|\alpha\right| , \tag{S14}$$

and

$$C_\mathrm{abs}(\omega) = k(\omega)\Im(\alpha) , \tag{S15}$$

where $\Im(\alpha)$ denotes the imaginary part of the polarizability.

By using the polarizability for a spherical particle (equation S1) and the expression for $\Gamma_{\mathrm{non-rad}}$ (equation S5), the radiative damping rate can be described by

$$\Gamma_{\mathrm{rad}} = \frac{k^3(\omega)}{6\pi} V \left(\frac{\epsilon_2(\omega)}{\epsilon_{\mathrm{m}}} + \frac{9\epsilon_{\mathrm{m}}}{\epsilon_2(\omega)} \right) \frac{2\hbar\epsilon_2(\omega)}{\left|\frac{d\epsilon_1(\omega)}{d\omega}\right|} \Bigg|_{\omega=\omega_{\mathrm{res}}} . \tag{S16}$$

For $1.4 < \hbar\omega < 2.2$, the imaginary part of the permittivity of gold is smaller than 2, meaning that the expression in the brackets of equation S16 is dominated by the second term. Further, $\Gamma_{\mathrm{non-rad}}$ is approximately constant, which gives the approximation

$$\Gamma_{\mathrm{rad}} = \frac{k^3(\omega_{\mathrm{res}})}{6\pi} V \frac{9\epsilon_{\mathrm{m}}}{\epsilon_2(\omega_{\mathrm{res}})} \Gamma_{\mathrm{non-rad}} = \frac{\omega_{\mathrm{res}}^3 \epsilon_2(\omega_0)}{\omega_0^3 \epsilon_2(\omega_{\mathrm{res}})} 2\hbar\kappa_{\mathrm{rad},0} V , \tag{S17}$$

with the radiation damping constant $\kappa_{\mathrm{rad},0}$ at the reference frequency ω_0. This yields a modified version of the often used radiation damping model[7,8]

$$\Gamma_{\mathrm{rad}} = 2\hbar\kappa_{\mathrm{rad}} V , \tag{S18}$$

that takes into account the frequency dependence of κ_{rad}, with $\kappa_{\mathrm{rad},0}$ still depending on the the permittivity of the surrounding medium. This simplified model for the plasmon damping in small gold spheres shows a simple dependence on the particle size and the resonance frequency, that allows straightforward comparison with experimental data to extract the empirical parameters A and $\kappa_{\mathrm{rad},0}$ for the nanoparticles. To do so, it is crucial to know the bulk values γ_{b}, ω_{p}, $\epsilon_2(\omega)$ and $\epsilon_{2,\mathrm{IB}}(\omega)$ of gold. The values of $\gamma_{\mathrm{b}} = 0.11\,\mathrm{fs}^{-1}$ and $\omega_{\mathrm{p}} = 13.7\,\mathrm{fs}^{-1}$ can be obtained from a fit of the Drude model[9] to experimentally measured data for the permittivity of gold.[5] The value of $\epsilon_{2,\mathrm{IB}}(\omega)$ can either be obtained by subtracting the Drude contribution from the experimentally obtained imaginary part of the dielectric permittivity[3] or from a full band-structure calculation.[10] Indeed, the results from both approaches agree very well, as shown in Figure S3(a). As plotted in Figure S3(b), the imaginary part of the

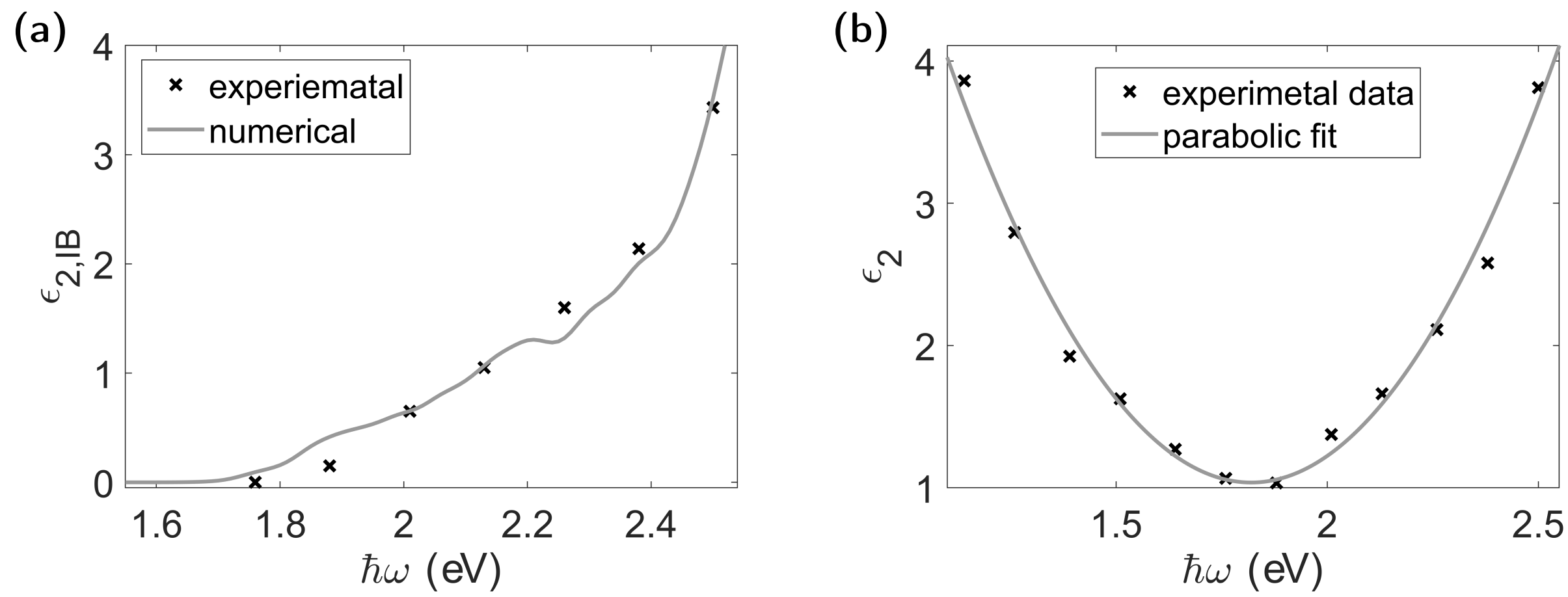

Figure S3: (a) Interband contribution to the imaginary part of the dielectric permittivity $\epsilon_{2,\mathrm{IB}}$ of gold, obtained from experimental data by subtracting the Drude contribution taken from Förster et al.[3] (symbols) and from numerical band structure calculations[10] (line, data kindly provided by Prof. Ravishankar Sundararaman, Rensselaer Polytechnic Institute). (b) Experimental data for the imaginary part of the dielectric permittivity ϵ_2 of gold (symbols),[5] fitted by a parabola with its minimum at $\hbar\omega_0 = 1.82\,\mathrm{eV}$ and $\epsilon_2(\omega_0) = 1.04$.

dielectric permittivity of gold is in the range of $1.1\,\mathrm{eV} < \hbar\omega < 2.5\,\mathrm{eV}$ well described by a parabola with its minimum at 1.82 eV. By choosing this value for ω_0, $\kappa_{\mathrm{rad},0}$ corresponds to the point of strongest radiation damping.

By further approximating the effective scattering length l_{eff} in the expression for surface damping by the particle length L parallel to the excitation polarization, the total damping in gold nanoparticles can be described by

$$\Gamma = \hbar\gamma_{\mathrm{b}} + \epsilon_{2,\mathrm{IB}}(\omega_{\mathrm{res}})\frac{\hbar\omega_{\mathrm{res}}^3}{\omega_{\mathrm{p}}^2} + \frac{\omega_{\mathrm{res}}^3\epsilon_2(\omega_0)}{\omega_0^3\epsilon_2(\omega_{\mathrm{res}})}2\hbar\kappa_{\mathrm{rad},0}V + \frac{\hbar\nu_{\mathrm{F}}A}{L}\,, \tag{S19}$$

with an expected value of $\kappa_{\mathrm{rad},0} \approx 4\cdot 10^{-7}\,\mathrm{fs}^{-1}\mathrm{nm}^{-3}$ for $\epsilon_{\mathrm{m}} = \epsilon_0$ according to equation S16, while A is in the range of 0 to 1. However, since the dielectric surrounding of the particles, which are placed on an ITO substrate and covered with QDs, is not homogeneous, the parameters $\kappa_{\mathrm{rad},0}$ and A shall be fitted to the experimental data, which can be done easily by varying the particle dimension perpendicular to the excitation polarization. This changes the volume and thus the radiation damping contribution, without effecting the other damping

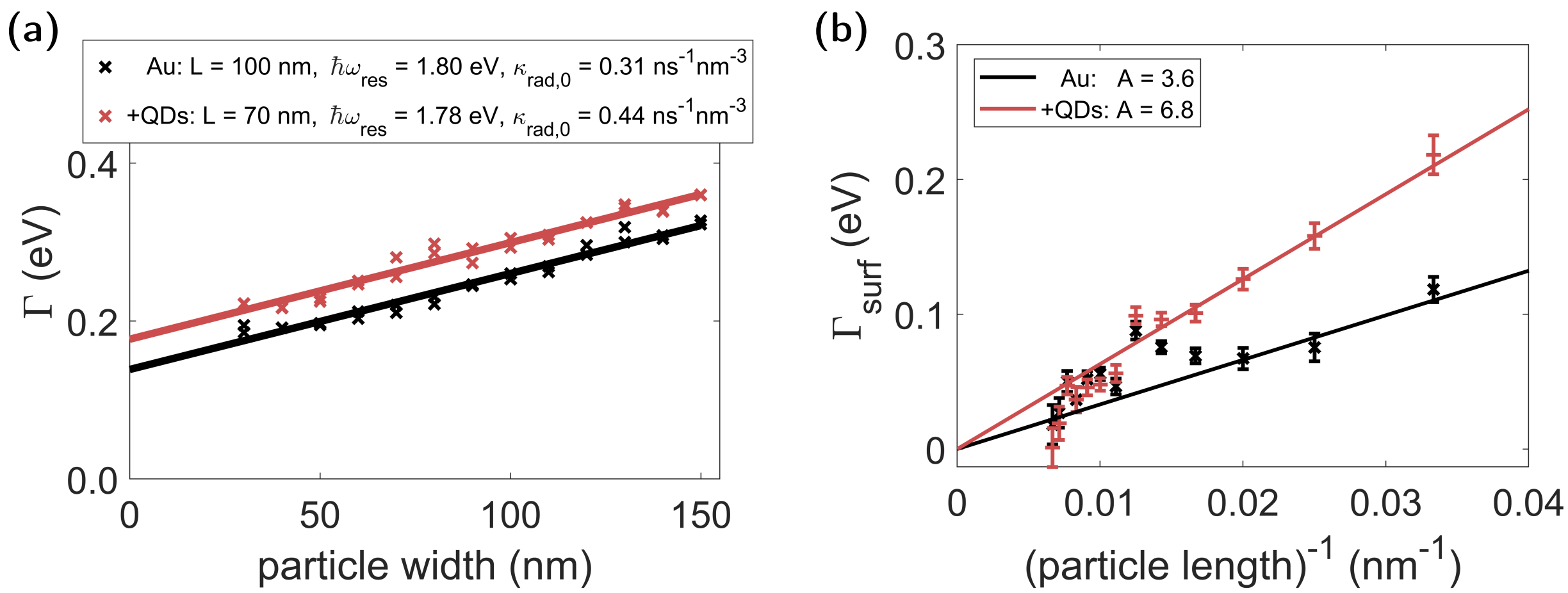

Figure S4: (a) Experimentally determined total damping Γ for rectangular gold nanocuboids (symbols), as a function of the particle width. The particle length parallel to the polarization direction is kept constant at 100 nm for uncovered gold particles and at 70 nm for QD-covered gold particles, the plasmon resonances are centered around 1.8 eV. The solid lines are a linear fit. From the slope of the fits the radiation damping constants as noted in the legend are derived. (b) Surface damping Γ_{surf} for uncovered and QD-covered particles (symbols), plotted with respect to to the inverse particle length L along the polarization direction. The solid lines are the respective fit functions.

terms. As shown in Figure S4(a), the experimental data confirm the predicted linear behavior of the damping on the particle volume, when the size parallel to the polarization is kept constant. By choosing now a particle set with $\omega_{\text{res}} \approx \omega_0$, the radiation damping constantan $\kappa_{\text{rad},0}$ can simply be determined from the slope of Γ with respect to the particle volume, as ϵ_2 is constant in first approximation. Uncovered particles with a length of 100 nm and QD-covered particles with a length of 70 nm parallel to the polarization fulfill this restriction. Radiation damping constants of $3.1 \cdot 10^{-7}\,\text{fs}^{-1}\text{nm}^{-3}$ and $4.4 \cdot 10^{-7}\,\text{fs}^{-1}\text{nm}^{-3}$ are derived for the bare and QD-covered particles, respectively, as shown in Figure S4(a). These values are close to the theoretically predicted value for a sphere and show the expected larger value for the QD-covered particles due to the increased permittivity of the surrounding medium. Following this argument the extrapolation of the fit towards zero particle width gives an approximation for the non-radiative damping part. The subsequent subtraction of the bulk damping and the interband damping allows finally for an estimation of the surface damping contribution, as plotted in Figure S4(b). From these data, we derive surface damping constants of 3.6 ± 0.8

and 6.8 ± 0.4 for the bare and the QD-covered particles, respectively. These values are significantly above the expected upper limit of 1 for surface damping, with might be due to the chosen approximation of $l_{\mathrm{eff}} \approx L$, the inhomogeneous environment[11] or a inhomogeneous field distribution in the rather large particles compared to particles where values below 1 are found.[12] The larger damping constant for the QD-covered particles indicates a chemical interface damping contribution.[13]

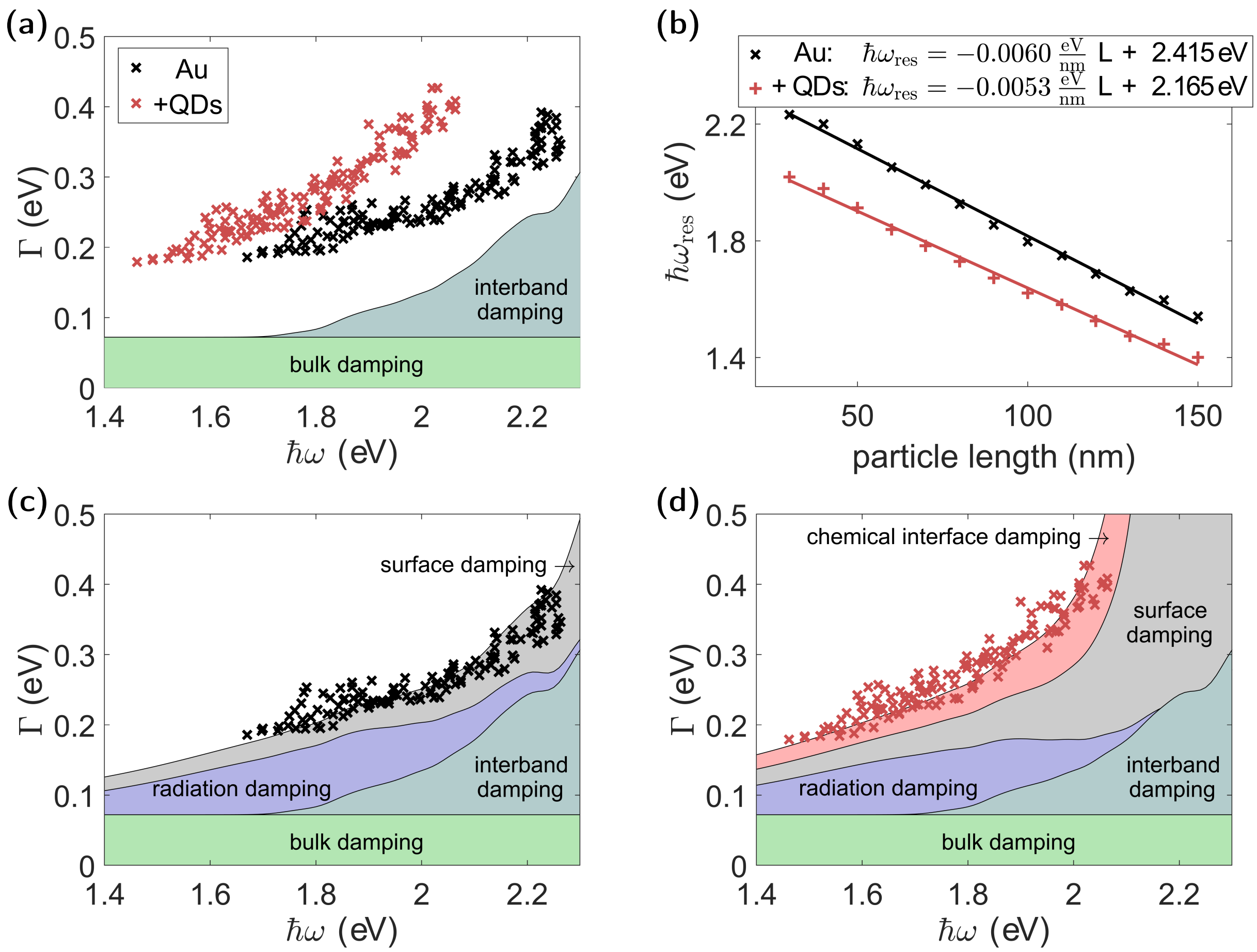

Figure S5: (a) Measured linewidth Γ with respect to the resonance energy $\hbar\omega$ of the set of uncovered (Au) and QD-covered (+QDs) gold particles (symbols) with the largest side length below 120 nm. The geometry-independent bulk and interband damping contributions are plotted according to the model discussed in the text. (b) Measured dependence of the resonance energy $\hbar\omega$ on the particle length L in polarization direction (symbols). The lines are linear fits. (c,d) Comparison of the measured linewidth Γ of uncovered and QD-covered gold particles with the full damping model by approximating the particle width with the average value of 70 nm.

The so derived model based on a small subset of the extinction measurements can now

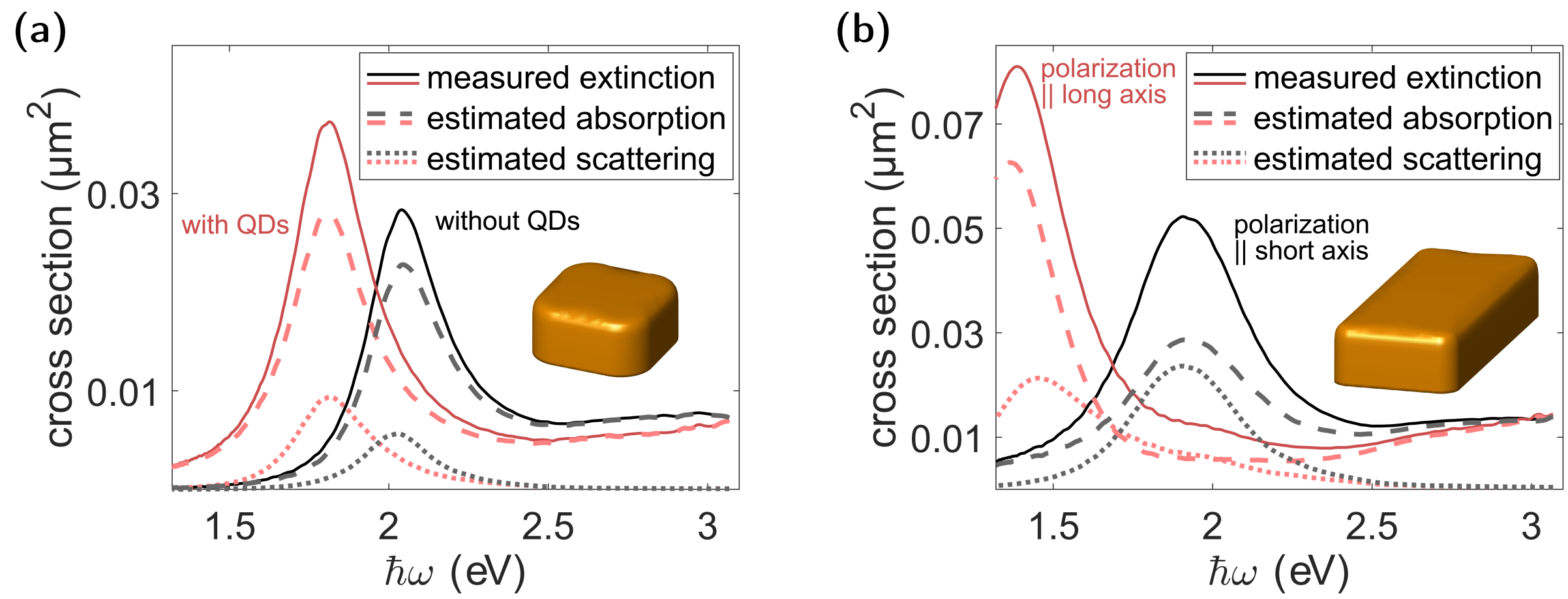

Figure S6: Absorption and scatting estimations based on the semi empiric model S19. In (a) for $60 \times 60 \times 30\,\mathrm{nm}^3$ gold particles with (red) and without (black) a QD layer on top. In (b) for $150 \times 60 \times 30\,\mathrm{nm}^3$ gold particles with a QD layer on top and polarization directions along the long axis (red) and the short axis (black) of the particle. The inserts illustrate the particle geometries.

be compared with a larger dataset. In Figure S5(a) the full linewidth data set for particles with the largest side length below 120 nm of uncovered and QD-covered particles is plotted, together with the geometry-independent bulk and interband damping contributions. To also include the geometry-dependent contributions in a compact way the fact that the resonance frequency depends only weakly on the particle size perpendicular to the polarization direction is exploited, as thus from the particle length in polarization direction the resonance frequency can be estimated, as depicted in Figure S5(b). Based on this relationship, each resonance energy can be assigned a particle length. With this and by approximating the particle width with the average value of 70 nm the full damping model describes the measured linewidths very well, as shown in Figure S5(c,d) for uncovered and QD-covered gold particles, respectively.

Using the relationship[14]

$$\frac{C_{\mathrm{sca}}(\omega)}{C_{\mathrm{abs}}(\omega)} = \frac{\Gamma_{\mathrm{rad}}}{\Gamma_{\mathrm{non-rad}}}\,, \tag{S20}$$

this model can now also be used to separate absorption and scattering contributions of a measured extinction spectrum and thus allow for an estimation of the scattering and absorp-

tion cross sections without the need for numerical calculation. In Figure S6(a) this is shown for a $60 \times 60 \times 30\,\mathrm{nm}^3$ uncovered (black) and QD-covered (red) gold particle. Figure S6(b) depicts the case of a $60 \times 150 \times 30\,\mathrm{nm}^3$ QD-covered gold particle, for the polarization directions along (red) and perpendicular (black) to the long particle axis. At 1.96 eV (633 nm) we find a ratio of 4.4 between the absorption cross sections for both polarization orientations. Accordingly, the value of 4.4 is used in the analysis of the photocurrents from gold nanoparticles in the main text. We note that the found ratios of absorption to scattering are similar to the ratios derived from Mie theory for spheres and ellipsoids of comparable size.[15]

S3 Energy level and thermionic emission model for a thin double Schottky barrier

In the following we develop a model for a thin double Schottky barrier system. By *thin* we mean that the semiconductor layer between two metal contacts is thinner than the depletion layer that would form at the contact between a single metal surface and an extended semiconductor. For a double Schottky barrier system with a thick semiconductor layer between two metal contacts, the two interfaces can be treated independently by a single Schottky barrier model each, as sketched in Figure S7(a). At both contacts, electrons from the semiconductor conduction band are transferred into the metal, building up a charge sheet at the metal surface and leaving back a depletion layer of thickness d_i in the semiconductor, which provides a potential barrier for charges from the interior of the semiconductor to the contacted surface. The associated band banding assures a vacuum level alignment of the metal/semiconductor interfaces. For a n-type semiconductor with a doping concentration N_d the width of the depletion layer d_i at the interface i is given by[16]

$$d_\mathrm{i} = \sqrt{\frac{2\varepsilon_\mathrm{sc}}{q_\mathrm{e} N_\mathrm{d}} \left(V_\mathrm{i} - V_\mathrm{ext}\right)}\,, \tag{S21}$$

with the permittivity of the semiconductor ε_sc, the elementary charge q_e, the applied voltage V_ext and the induced potential barrier on the semiconductor side V_i. The potential barrier V_i is given by the relationship

$$q_\mathrm{e}\Phi_\mathrm{B,i} = q_\mathrm{e}\left(\Phi_\mathrm{i} - X\right) = q_\mathrm{e}V_\mathrm{i} + \left(E_\mathrm{F} - E_\mathrm{CBM}\right)\,, \tag{S22}$$

with the Schottky barrier $\Phi_\mathrm{B,i}$, the work function of metal i Φ_i, the electron affinity of the semiconductor X and the difference between the Fermi level and the semiconductor conduction band minimum $E_\mathrm{F} - E_\mathrm{CBM}$.

When the semiconductor layer becomes thinner than the sum of the widths of the two

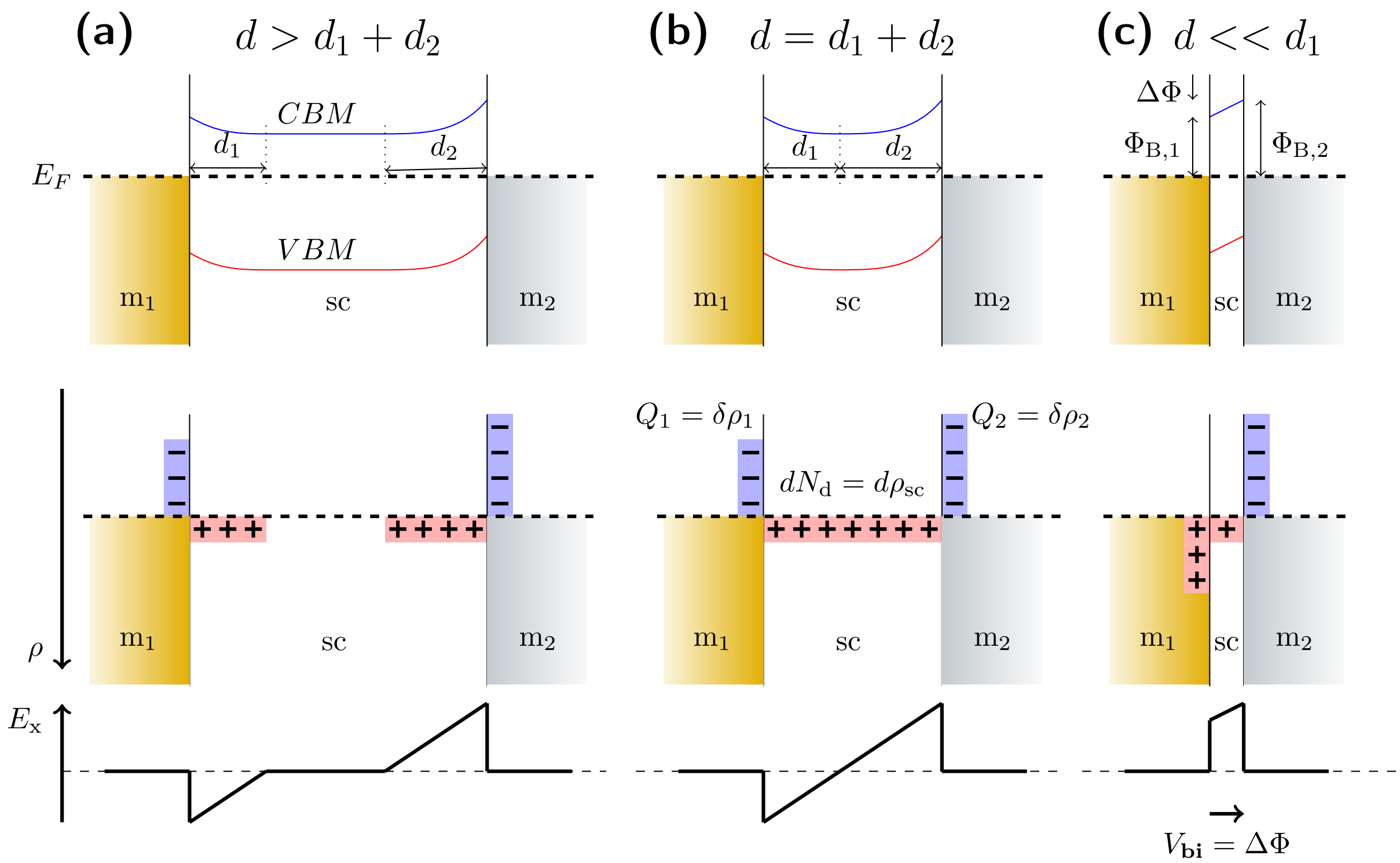

Figure S7: Sketches of energy levels of the conduction band minimum (CBM) and the valance band maximum (VBM), the charge density ρ and the horizontal electric field E_x in a double Schottky barrier system. The semiconductor layer thicknesses d is assumed to be (a) larger than, (b) equal to and (c) smaller than the sum of the single interface depletion layer thickness d_1 and d_2. The Schottky barriers at the two metal m_1 and m_2 interfaces with the semiconductor layer sc are $\Phi_{B,1}$ and $\Phi_{B,2}$, their difference $\Delta\Phi$ corresponds to the built-in voltage V_{bi} in case (c). The semiconductor layer is assumed to be n-doped with a concentration N_d equal to the charge density in the depleted areas. The accumulated surface charges at the electrodes Q_1 and Q_2 are assumed to be finitely extended within a width δ, with the charge densities ρ_1 and ρ_2.

depletion layers ($d < d_1 + d_2$) the semiconductor is fully depleted and the two interfaces cannot be treated independently any more. The semiconductor provides a fixed amount of electrons dN_d, but in order to further ensure vacuum alignment at the two interfaces, additional charge transfer between the metal surfaces might be necessary to account for a potential work function difference between them. The system that needs to be considered is sketched in Figure S7, giving an electric field E of

$$\begin{aligned} E(x) &= \lim_{\delta\to 0}\int_{-d/2-\delta}^{x} \frac{\rho(\xi)}{2\varepsilon_\mathrm{sc}}\,d\xi - \lim_{\delta\to 0}\int_{x}^{d/2+\delta} \frac{\rho(\xi)}{2\varepsilon_\mathrm{sc}}\,d\xi \\ &= \lim_{\delta\to 0}\int_{-d/2-\delta}^{-d/2} \frac{Q_1}{\delta 2\varepsilon_\mathrm{sc}}\,d\xi + \int_{-d/2}^{x} \frac{\rho_\mathrm{sc}}{2\varepsilon_\mathrm{sc}}\,d\xi - \int_{x}^{d/2} \frac{\rho_\mathrm{sc}}{2\varepsilon_\mathrm{sc}}\,d\xi - \lim_{\delta\to 0}\int_{d/2}^{d/2+\delta} \frac{Q_2}{\delta 2\varepsilon_\mathrm{sc}}\,d\xi \\ &= \frac{Q_1 - Q_2}{2\varepsilon_\mathrm{sc}} + \frac{\rho_\mathrm{sc}}{\varepsilon_\mathrm{sc}}x\,, \end{aligned} \tag{S23}$$

for x inside the semiconductor, with the charge density distribution ρ, the charge density $\rho_\mathrm{sc} = q_\mathrm{e}N_\mathrm{d}$ in the semiconductor and the surface charge densities Q_i at the metal surface i. To ensure overall charge neutrality the condition

$$Q_1 + Q_2 + d\rho_\mathrm{sc} = 0\,, \tag{S24}$$

must hold. The potential $\Phi(x)$ for a position $x \in (-d/2, d/2)$ inside the semiconductor is then given by

$$\Phi(x) = -\int_{-d/2}^{x} E(\xi)\,d\xi = \frac{d\,(Q_2 - Q_1)}{4\varepsilon_\mathrm{sc}}\left(1 + \frac{2x}{d}\right) + \frac{d^2\rho_\mathrm{sc}}{8\varepsilon_\mathrm{sc}}\left(1 - \left(\frac{2x}{d}\right)^2\right). \tag{S25}$$

To correctly account for vacuum aliment at the two interfaces, the condition

$$\frac{d\,(Q_2 - Q_1)}{2\varepsilon_\mathrm{sc}} = \Phi_2 - \Phi_1 =: \Delta\Phi\,, \tag{S26}$$

must hold. Consequently, we we can determine the surface charge densities as

$$Q_1 = -\frac{d}{2} q_\mathrm{e} N_\mathrm{d} - \frac{\Delta\Phi \varepsilon_\mathrm{sc}}{d} , \tag{S27}$$

$$Q_2 = -\frac{d}{2} q_\mathrm{e} N_\mathrm{d} + \frac{\Delta\Phi \varepsilon_\mathrm{sc}}{d} , \tag{S28}$$

and finally we get

$$\Phi(x) = \frac{\Delta\Phi}{2} \left(1 + \frac{2x}{d}\right) + \frac{d^2 \rho_\mathrm{sc}}{8\varepsilon_\mathrm{sc}} \left(1 - \left(\frac{2x}{d}\right)^2\right) , \tag{S29}$$

showing that the band curvature in the semiconductor becomes negligible for very thin films with $d << d_1 + d_2$, as schematically shown in Figure S7 (c). The flattening of the band structure means that the electric field between two electrodes with different work functions shows only one sign and becomes almost constant, as sketched in Figure S7 (c). Therefore, excited charge carriers are always accelerated in the direction of a specific electrode, in contrast to the case of separated depletion layers, in which the transport direction depends on the position of the charge carrier in the semiconductor layer. Consequently, a very thin film can be meaningfully described by a single built-in voltage V_bi across the entire layer, equal to the work function difference $\Delta\Phi$ of the two electrodes. We note here that surface-induced localized gap states confined to a fraction of the semiconductor layer thickness can alter this picture by forming two separated electrical double layers at the metal-semiconductor interfaces. A model also including potential Fermi level pinning is given in section S3.1, with the main difference that the heights of the Schottky barriers can decouple from the work functions.

From the energy level scheme from Figure S7 (c) we can deduce a current / voltage model based on thermionic emission. For an externally applied bias voltage V_ext we preliminary distinguish between the the cases $V_\mathrm{ext} < -V_\mathrm{bi}$ and $V_\mathrm{ext} > -V_\mathrm{bi}$, as shown in Figure S8. Considering only electron transport, in the case of $V_\mathrm{ext} < -V_\mathrm{bi}$ the barrier $\Phi_{1\rightarrow 2}$ for an

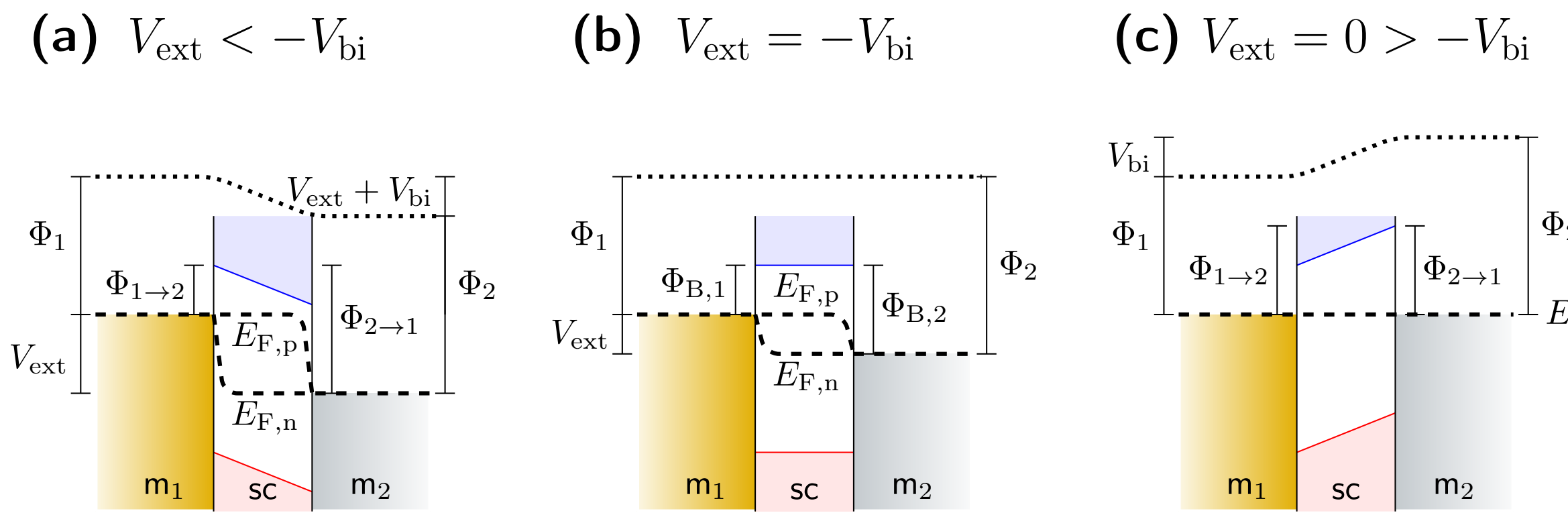

Figure S8: Sketches of the bias-dependent energy level diagrams of an ultrathin double Schottky barrier. The diagrams assume the work function Φ_2 of the metal electrode m_2 to be larger than the work function Φ_1 of the metal electrode m_1, giving a positive built-in voltage $V_{\rm bi}$ across the very thin semiconductor layer sc. (a) Applied bias voltage $V_{\rm ext}$ smaller than $-V_{\rm bi}$. The quasi-Fermi-level of electrons $E_{\rm F,n}$ in the semiconductor layer lies at the Fermi level $E_{\rm F}$ of m_2 and the quasi-Fermi-level of holes $E_{\rm F,p}$ lies at the Fermi level of m_1. (b) $V_{\rm ext}$ equal to $-V_{\rm bi}$, where the barrier $\Phi_{1\to2}$ for an electron to move from m_1 to m_2 is equal to the single contact barrier $\Phi_{\rm B,1}$ and the barrier $\Phi_{2\to1}$ for an electron to move from m_2 to m_1 is equal to the single contact barrier $\Phi_{\rm B,2}$. (c) $V_{\rm ext}=0$, corresponding to $V_{\rm ext}$ greater than $-V_{\rm bi}$. The vacuum levels are indicated by the dotted lines.

electron to move from electrode 1 to electrode 2 is given by $\Phi_{\rm B,1}$ and the barrier $\Phi_{2\to1}$ for an electron to move from electrode 2 to electrode 1 is given by $\Phi_{\rm B,1}-V_{\rm ext}$. Consequently, the thermionic emission current $I_{\rm th}$ in the first case is given by

$$I_{\rm th}(V_{\rm ext} < -V_{\rm bi}) = FA^*T^2 \exp\left(-\frac{e\Phi_{\rm B,1}}{k_{\rm B}T}\right)\left(\exp\left(\frac{eV_{\rm ext}}{k_{\rm B}T}\right)-1\right), \tag{S30}$$

with the electrode area F, the Richardson constant A^*, the elementary charge e, the temperature T and the Boltzmann constant $k_{\rm B}$. For the case of $V_{\rm ext} > -V_{\rm bi}$ the barrier $\Phi_{1\to2}$ for an electron to move from electrode 1 to electrode 2 is given by $\Phi_{\rm B,2}+V_{\rm ext}$ and the barrier $\Phi_{2\to1}$ for an electron to move from electrode 2 to electrode 1 is given by $\Phi_{\rm B,2}$. Consequently the thermionic emission current in the second case $I_{\rm th}$ is given by

$$I_{\rm th}(V_{\rm ext} > -V_{\rm bi}) = -FA^*T^2 \exp\left(-\frac{e\Phi_{\rm B,2}}{k_{\rm B}T}\right)\left(\exp\left(-\frac{eV_{\rm ext}}{k_{\rm B}T}\right)-1\right). \tag{S31}$$

Combining equations S30 and S31 we get

$$I_{\mathrm{th}} = -sFA^*T^2 \exp\left(-\frac{e\tilde{\Phi}_{\mathrm{s}}}{k_{\mathrm{B}}T}\right)\left(\exp\left(-\frac{esV_{\mathrm{ext}}}{k_{\mathrm{B}}T}\right) - 1\right) , \tag{S32}$$

with $s := \mathrm{sign}\,(V_{\mathrm{ext}} + V_{\mathrm{bi}})$, $\tilde{\Phi}_{-1} := \Phi_{\mathrm{B},1}$ and $\tilde{\Phi}_{+1} := \Phi_{\mathrm{B},2}$, resulting in a classical thermionic emission equation for a single Schottky barrier biased in blocking direction. To take into account the dependence of the potential barrier height on the applied bias due to mirror charges and the generation/recombination in the depleted semiconductor we introduce the ideality factor n_s in the commonly used representation[16,17] as

$$\frac{1}{n_s} = 1 - \left.\frac{\partial\tilde{\Phi}_s}{\partial V}\right|_{V\to 0^-} , \tag{S33}$$

substitute the expression

$$\tilde{\Phi}_s = \Phi_s - \left(1 - \frac{1}{n_{\mathrm{sign}(V_{\mathrm{ext}})}}\right)|V_{\mathrm{ext}}| , \tag{S34}$$

in equation S32 and arrive at

$$I_{\mathrm{th}} = -sFA^*T^2\Delta\left(V_{\mathrm{ext}}\right) \exp\left(-\frac{e\Phi_s}{k_{\mathrm{B}}T}\right) \exp\left(-\frac{esV_{\mathrm{ext}}}{n_{\mathrm{sign}(V_{\mathrm{ext}})}k_{\mathrm{B}}T}\right)\left(1 - \exp\left(\frac{esV_{\mathrm{ext}}}{k_{\mathrm{B}}T}\right)\right) , \tag{S35}$$

with

$$\Delta\left(V_{\mathrm{ext}}\right) = \exp\left(-\frac{e\left(n_{\mathrm{sign}(V_{\mathrm{ext}})} - 1\right)\left(sV_{\mathrm{ext}} - |V_{\mathrm{ext}}|\right)}{n_{\mathrm{sign}(V_{\mathrm{ext}})}k_{\mathrm{B}}T}\right), \tag{S36}$$

which coincides for the case of $\Phi_{\mathrm{B},1} = \Phi_{\mathrm{B},2}$ with the description obtained in a previous study assuming a 2-dimensional semiconductor layer.[18] When also including partial Fermi level pinning we have to adapt the expression for the sign by $s := \mathrm{sign}\,(V_{\mathrm{ext}} + \Phi_{\mathrm{B},2} - \Phi_{\mathrm{B},1})$.

S3.1 Extension to Fermi level pinning

Assuming surface-induced gap states with an extension of length a into the semiconductor, Figure S7 can be extended as shown in Figure S9. For a being of atomic length scale ($< 1\,\mathrm{nm}$), electron tunneling is very efficient, e.g., the transmission probability is about 0.3 for a barrier height of 1 eV (0.5 eV), a barrier width of 0.3 nm and a kinetic energy of the electron of 0.5 eV (0.1 eV). Essentially, the energy barrier for thermionic emission is thus given by the difference of the Fermi level and the CBM at the distance a inside the semiconductor, which is independent of the metal work function in the case of full Fermi level pinning.[19–21] The electric field in such a system is given by

$$E(x) = \lim_{\delta\to 0}\int_{-d/2-\delta}^{x} \frac{\rho(\xi)}{2\varepsilon_{\mathrm{sc}}}\,d\xi - \lim_{\delta\to 0}\int_{x}^{d/2+\delta} \frac{\rho(\xi)}{2\varepsilon_{\mathrm{sc}}}\,d\xi$$
$$= \begin{cases} \frac{Q_1}{\varepsilon_{\mathrm{sc}}} + \frac{Q_{\mathrm{g},1}}{2\varepsilon_{\mathrm{sc}}a}\left(2x+d\right), & x\in\left(-\frac{d}{2}, -\frac{d}{2}+a\right) \\ \frac{2Q_1+2Q_{\mathrm{g},1}+\rho_{\mathrm{sc}}(d-2a)}{2\varepsilon_{\mathrm{sc}}} + x\frac{\rho_{\mathrm{sc}}}{\varepsilon_{\mathrm{sc}}}, & x\in\left(-\frac{d}{2}+a, \frac{d}{2}-a\right) \\ -\frac{Q_2}{\varepsilon_{\mathrm{sc}}} + \frac{Q_{\mathrm{g},2}}{2\varepsilon_{\mathrm{sc}}a}\left(d-2x\right), & x\in\left(\frac{d}{2}-a, \frac{d}{2}\right) \end{cases}, \tag{S37}$$

where we used the overall charge neutrality condition

$$Q_1 + Q_{\mathrm{s},1} + \rho_{\mathrm{sc}}(d-2a) + Q_{\mathrm{s},2} + Q_2 = 0\,. \tag{S38}$$

This gives an electrostatic potential of

$$\Phi(x) = -\int_{-d/2}^{x} E(\xi)\,d\xi$$
$$= \begin{cases} 0, & x\in\left(-\infty, -\frac{d}{2}\right) \\ -\frac{Q_1}{2\varepsilon_{\mathrm{sc}}}\left(2x+d\right) - \frac{Q_{\mathrm{g},1}}{8\varepsilon_{\mathrm{sc}}a}\left(2x+d\right)^2, & x\in\left(-\frac{d}{2}, -\frac{d}{2}+a\right) \\ -\frac{2Q_1+Q_{\mathrm{g},1}}{2\varepsilon_{\mathrm{sc}}}a - \frac{Q_1+Q_{\mathrm{g},1}}{2\varepsilon_{\mathrm{sc}}}\left(2x+d-2a\right) - \frac{\rho_{\mathrm{sc}}}{8\varepsilon_{\mathrm{sc}}}\left(2x+d-2a\right)^2, & x\in\left(-\frac{d}{2}+a, \frac{d}{2}-a\right) \\ -\frac{Q_1+Q_{\mathrm{g},1}-Q_{\mathrm{g},2}-Q_2}{2\varepsilon_{\mathrm{sc}}}d + \frac{Q_{\mathrm{g},1}-Q_{\mathrm{g},2}}{2\varepsilon_{\mathrm{sc}}}a + \frac{Q_2}{2\varepsilon_{\mathrm{sc}}}\left(2x-d\right) - \frac{Q_{\mathrm{g},2}}{8\varepsilon_{\mathrm{sc}}a}\left(2x-d\right)^2, & x\in\left(\frac{d}{2}-a, \frac{d}{2}\right) \\ \frac{Q_2-Q_1}{2\varepsilon_{\mathrm{sc}}}d + \frac{Q_{\mathrm{g},2}-Q_{\mathrm{g},1}}{2\varepsilon_{\mathrm{sc}}}(d-a), & x\in\left(\frac{d}{2}, \infty\right) \end{cases}, \tag{S39}$$

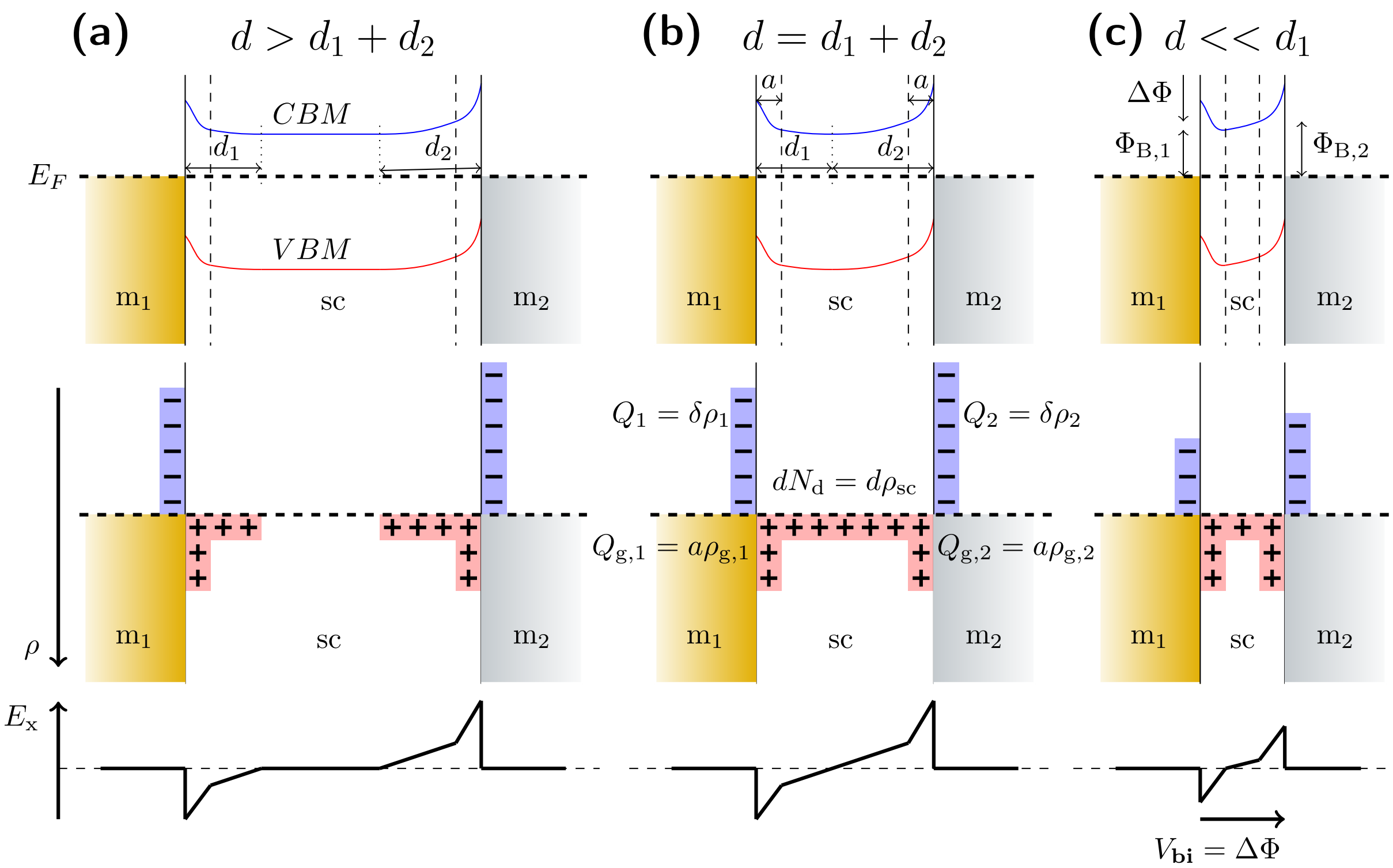

Figure S9: Sketches of the energy levels of the conduction band minimum (CBM) and the valance band maximum (VBM), the charge density ρ and the horizontal electric field E_x in a double Schottky barrier system with Fermi-level pining included by surface-induced gap states, with an extension of length a into the semiconductor, accumulating charges of amount $Q_{g,1}$ and $Q_{g,2}$ determined by the charge densities $\rho_{g,1}$ and $\rho_{g,2}$. The cases are shown for a semiconductor layer thicknesses d that is (a) larger than, (b) equal to and (c) smaller than the sum of the single interface depletion layer thicknesses d_1 and d_2. The Schottky barriers $\Phi_{B,1}$ and $\Phi_{B,2}$ at the two interfaces are given by the potential difference between metal surfaces $m_{1/2}$ and the CBM at the distance a inside the semiconductor layer sc, with a difference of $\Delta\Phi$. The work function difference of the two metal electrodes m_1 and m_2 equals the built-in voltage V_{bi} in case (c). The semiconductor layer is assumed to be n-doped with a doping concentration N_d equal to the charge density in the depleted areas. The accumulated surface charges at the electrodes Q_1 and Q_2 are assumed to be finitely extended with a width δ and charge densities ρ_1 and ρ_2.

thus the potential drop across the two surface-induced gap state regions is given by $(-1)^i(2Q_i + Q_{\mathrm{g},i})a/(2\varepsilon_{\mathrm{sc}})$ for $i \in (1,2)$. For the case of full Fermi level pinning the potential drop at the the two surface-induced gap state regions must equal the work function difference between the metal and the semiconductor, giving

$$2Q_1 + Q_{\mathrm{g},1} = -\frac{2\varepsilon_{\mathrm{sc}}}{a}\Delta\Phi_1\,, \tag{S40}$$

$$2Q_2 + Q_{\mathrm{g},2} = \frac{2\varepsilon_{\mathrm{sc}}}{a}\Delta\Phi_2\,, \tag{S41}$$

with $\Delta\Phi_1 = (X + (E_{\mathrm{CBM}} - E_{\mathrm{F}}^*)/q_{\mathrm{e}} - \Phi_1)$ and $\Delta\Phi_2 = (\Phi_2 - X - (E_{\mathrm{CBM}} - E_{\mathrm{F}}^*)/q_{\mathrm{e}})$ and E_{F}^* the Fermi level at the pinned surface. Further, the potential drop over the total double layer $\Phi(d/2)$ has to compensate for the work function difference $\Delta\Phi = \Phi_2 - \Phi_1$ of the two metals, giving

$$2\varepsilon_{\mathrm{sc}}\Delta\Phi = (Q_2 - Q_1)d + (Q_{\mathrm{g},2} - Q_{\mathrm{g},1})(d-a)\,. \tag{S42}$$

The resulting system of equations S38, S40, S41 and S42 can be solved for the four unknown, leading to

$$Q_1 = \frac{\rho_{\mathrm{sc}}}{2}(d-2a) - \frac{2\varepsilon_{\mathrm{sc}}}{a}\Delta\Phi_1\,, \tag{S43}$$

$$Q_2 = \frac{\rho_{\mathrm{sc}}}{2}(d-2a) + \frac{2\varepsilon_{\mathrm{sc}}}{a}\Delta\Phi_2\,, \tag{S44}$$

$$Q_{\mathrm{g},1} = -\rho_{\mathrm{sc}}(d-2a) + \frac{2\varepsilon_{\mathrm{sc}}}{a}\Delta\Phi_1\,, \tag{S45}$$

$$Q_{\mathrm{g},2} = -\rho_{\mathrm{sc}}(d-2a) - \frac{2\varepsilon_{\mathrm{sc}}}{a}\Delta\Phi_2\,. \tag{S46}$$

To account for partial Fermi level pinning we introduce the parameter S^* for a linear transition of $Q_{\mathrm{g},1}$ from full Fermi level pinning ($S^* = 0$) to full vacuum level alignment ($S^* = 1$) with a carrier density equal to the interior of the semiconductor ρ_{sc}. This leads to

$$Q_{\mathrm{g},1}(S^*) = -\rho_{\mathrm{sc}}(d - 2a - S^*(d-a)) + (1-S^*)\frac{2\varepsilon_{\mathrm{sc}}}{a}\Delta\Phi_1\,, \tag{S47}$$

$$Q_{\mathrm{g},2}(S^*) = -\rho_{\mathrm{sc}}(d-2a-S^*(d-a)) - (1-S^*)\frac{2\varepsilon_{\mathrm{sc}}}{a}\Delta\Phi_2\,. \tag{S48}$$

Inserting these new conditions for $Q_{\mathrm{g},1}(S^*)$ and $Q_{\mathrm{g},2}(S^*)$ into equations S38 and S42, we get

$$Q_1(S^*) = -\frac{\rho_{\mathrm{sc}}}{2}S^*(d-a) - (1-S^*)\frac{2\varepsilon_{\mathrm{sc}}}{a}\Delta\Phi_1 - S^*\frac{\varepsilon_{\mathrm{sc}}}{d}\Delta\Phi\,, \tag{S49}$$

$$Q_2(S^*) = -\frac{\rho_{\mathrm{sc}}}{2}S^*(d-a) + (1-S^*)\frac{2\varepsilon_{\mathrm{sc}}}{a}\Delta\Phi_2 + S^*\frac{\varepsilon_{\mathrm{sc}}}{d}\Delta\Phi\,. \tag{S50}$$

For the resulting dependence of the Schottky barrier height on the parameter S^* we get

$$\begin{aligned}\Phi_{\mathrm{B},1}(S^*) &= \Phi_1 - X - \frac{a}{2\varepsilon_{\mathrm{sc}}}\left(2Q_1(S^*) + Q_{\mathrm{g},1}(S^*)\right)\\ &= -\Delta\Phi_1 - \frac{a}{2\varepsilon_{\mathrm{sc}}}\left(2Q_1(S^*) + Q_{\mathrm{g},1}(S^*)\right) + (E_{\mathrm{CBM}} - E_{\mathrm{F}}^*)/q_{\mathrm{e}}\\ &= -S^*\Delta\Phi_1 + S^*\frac{a}{d}\Delta\Phi + \frac{a}{2\varepsilon_{\mathrm{sc}}}\rho_{\mathrm{sc}}(d-a) + (E_{\mathrm{CBM}} - E_{\mathrm{F}}^*)/q_{\mathrm{e}}\,,\end{aligned} \tag{S51}$$

$$\begin{aligned}\Phi_{\mathrm{B},2}(S^*) &= \Phi_2 - X - \frac{a}{2\varepsilon_{\mathrm{sc}}}\left(2Q_2(S^*) + Q_{\mathrm{g},2}(S^*)\right)\\ &= \Delta\Phi_2 - \frac{a}{2\varepsilon_{\mathrm{sc}}}\left(2Q_1(S^*) + Q_{\mathrm{g},1}(S^*)\right) + (E_{\mathrm{CBM}} - E_{\mathrm{F}}^*)/q_{\mathrm{e}}\\ &= S^*\Delta\Phi_2 - S^*\frac{a}{d}\Delta\Phi + \frac{a}{2\varepsilon_{\mathrm{sc}}}\rho_{\mathrm{sc}}(d-a) + (E_{\mathrm{CBM}} - E_{\mathrm{F}}^*)/q_{\mathrm{e}}\,.\end{aligned} \tag{S52}$$

Partial Fermi level pinning is typically described by the slope S (Ref. [22,23]) that is defined by the partial derivative of the Schottky barrier height with respect to the metal work function. For the case discussed above we find for $i \in (1,2)$

$$S = \frac{\partial\Phi_{\mathrm{B},i}(S^*)}{\partial\Phi_i} = \left(1 - \frac{a}{d}\right)S^*\,, \tag{S53}$$

giving a simple relationship between the here introduced linear transition parameter S^* for the charges in the surface-induced gap state regions and the parameter S describing the dependence of the Schottky barrier height on the metal work function. It should be noted that the slight discrepancy between S and S^* arises from the fact that for the surface-induced gap states a finite extension into the semiconductor was chosen in the model and

the Schottky barrier height is measured at this distance a inside the semiconductor. In the case of full vacuum alignment $S^* \rightarrow 1$ the distance a looses its physical meaning and $a \rightarrow 0$ is required, meaning that S^* and S coincide at full vacuum alignment and full Fermi pinning. In between, the necessity of a finite distance between the measurement point of the Schottky barrier height and the metal surface gives the observed difference between surface-induced gap state charge variation S^* and the variation S of the Schottky barrier height with respect to the metal work function.

S4 Energy levels of $PbS/MAPbI_3$ QDs

The absorption spectrum of QDs is given by a superposition of absorption peaks corresponding to the confinement energies,[24] typically Gaussian-broadened due to QD size dispersion. Thus, the second derivative of the absorption spectrum can be used to increase the feature resolution and identify electronic transitions.[25] In Figure S10(a) the second derivative of the absorption spectrum measured from $PbS/MAPbI_3$ QDs in a solution (red) and from a monolayer on ITO (black) are shown. Local minima that appear in both measurements are indicated with lowercase Greek letters, their energy values are listed in table S1. Cademartiri et al.[26] determined the transition energies for oleic-acid-capped PbS QDs with diameters from 4 nm to 7 nm, the extrapolation of their findings to a diameter of 3.2 nm are listed in table S1. Our findings are in very good agreement with the data of Cademartiri et al. (except for β), indicating that the energy levels in the used $PbS/MAPbI_3$ QDs are very similar to those of oleic-acid-capped PbS QDs. Further a good agreement with the prediction from the hyperbolic band model (HBM)[27] is observed as shown in table S1.

Table S1: Experimentally observed transitions in $PbS/MAPbI_3$ QDs compared with literature data of PbS QDs and transition energies of PbS QDs in the hyperbolic band model (HBM).

min.	name of the local minimum as observed in Figure S10 (a)
sol.	position of the local minimum measured in solution, see Figure S10 (a)
mono.	position of the local minimum measured as monolayer, see Figure S10 (a)
PL	photoluminescence peak maximum as observed in Figure S10 (b)
lit.	extrapolation of the data from Ref. [26] to a QD diameter of 3.2 nm
HBM	transition energies predicted by the hyperbolic band model[27]
transition	corresponding transition in the hyperbolic band model[28]

min.	sol. (eV)	mono. (eV)	PL (eV)	lit.	HBM (eV)	transition
α	1.27	1.27	1.29	1.32	1.28	$1S_h \rightarrow 1S_e$
β	1.47	1.43	1.41			
γ	1.59	1.61		1.63	1.62	$1S_h \rightarrow 1P_e$ ($1P_h \rightarrow 1S_e$)
δ	1.77	1.82		1.76		
ϵ	1.96	1.96		1.90	1.97	$1P_h \rightarrow 1P_e$
ζ	2.26	2.26		2.20		
ω		1.24	1.24			

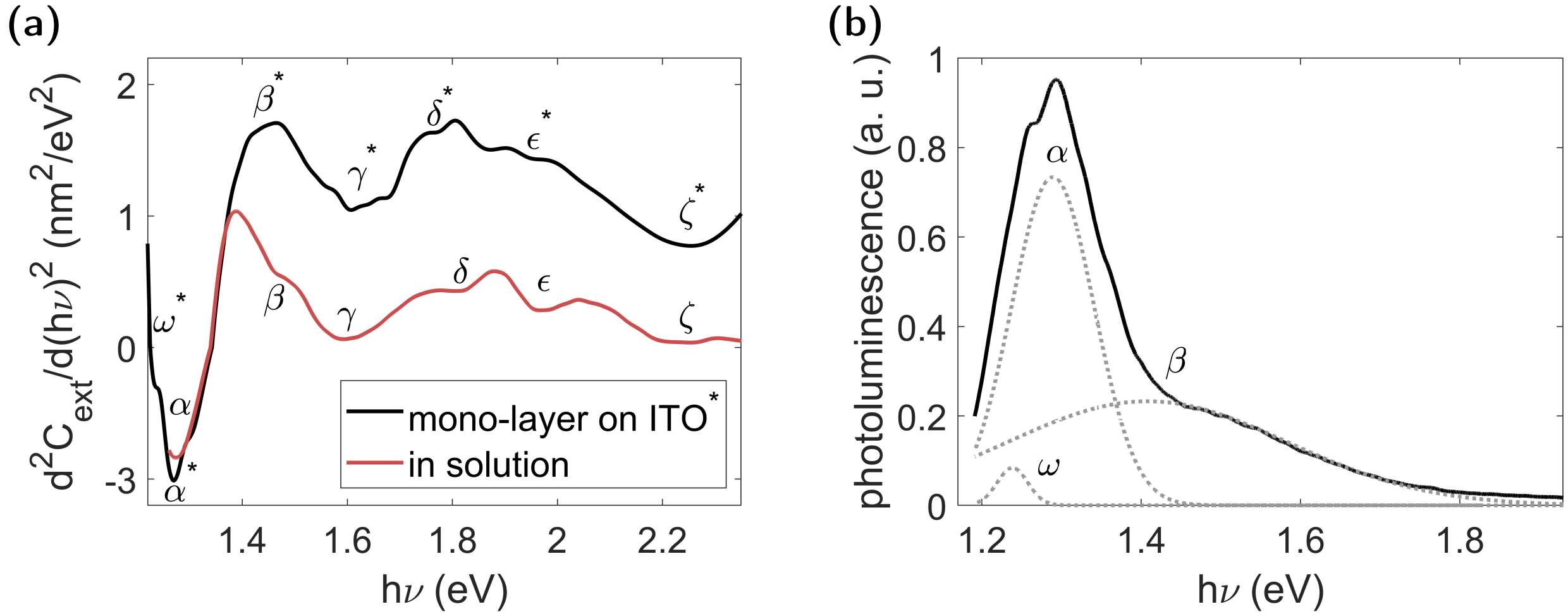

Figure S10: $PbS/MAPbI_3$ energy levels. (a) Second derivative of the absorption spectrum of $PbS/MAPbI_3$ QDs, measured in solution (red) and from a monolayer on ITO (black). The measurements were smoothed using a moving average over a wavelength interval of 25 nm, corresponding approximately to an energy interval of 0.06 eV. Local minima are named with lowercase Greek letters. Letters with an asterisk indicate minima in the measurement at the monolayer that are assigned minimum in the data acquired from solution. (b) Photoluminescence from $PbS/MAPbI_3$ QDs in solution, the dotted lines give the three Gaussian functions which describe the measured spectrum ($\mu_\omega = 1.24\,\text{eV}$, $\sigma_\omega = 0.02\,\text{eV}$, $\mu_\alpha = 1.29\,\text{eV}$, $\sigma_\alpha = 0.05\,\text{eV}$, $\mu_\beta = 1.41\,\text{eV}$, $\sigma_\beta = 0.18\,\text{eV}$).

In contrast to the PbS core with a large exciton Bohr radius[29,30] of 10 nm ($m^* \approx 0.09\,m_0$, Ref.,[29] $\epsilon_r \approx 17$, Ref.[29]), the $MAPbI_3$ shell has a small exciton Bohr radius of about 1 nm ($m^* \approx 0.3m_0$, Ref.,[31] $\epsilon_r \approx 6$, Ref.[32]). Consequently, the quantum confinement has very little effect on the $MAPbI_3$ energy levels, as evidenced by just small spectral shifts of the luminescence peaks of $MAPbI_3$ QDs with respect to bulk $MAPbI_3$,[33] and by the similar absorption spectra of the bulk material[32] and QDs,[34] with an absorption onset around 800 nm (1.55 eV).

In the second derivative function in Figure S10(a), feature β resembles rather a saddle point than a minimum, which might indicate the absorption onset of the $MAPbI_3$ shell. This is in agreement with the photoluminescence spectrum in Figure S10(b), where we find in addition to the PbS fluorescence peak at 1.29 eV a broad peak at 1.41 eV, which are revealed by fitting a sum of Gaussians, as shown in Figure S10(b). Concerning the third peak ω in the photoluminescence spectrum we can also identify a feature in the second derivative

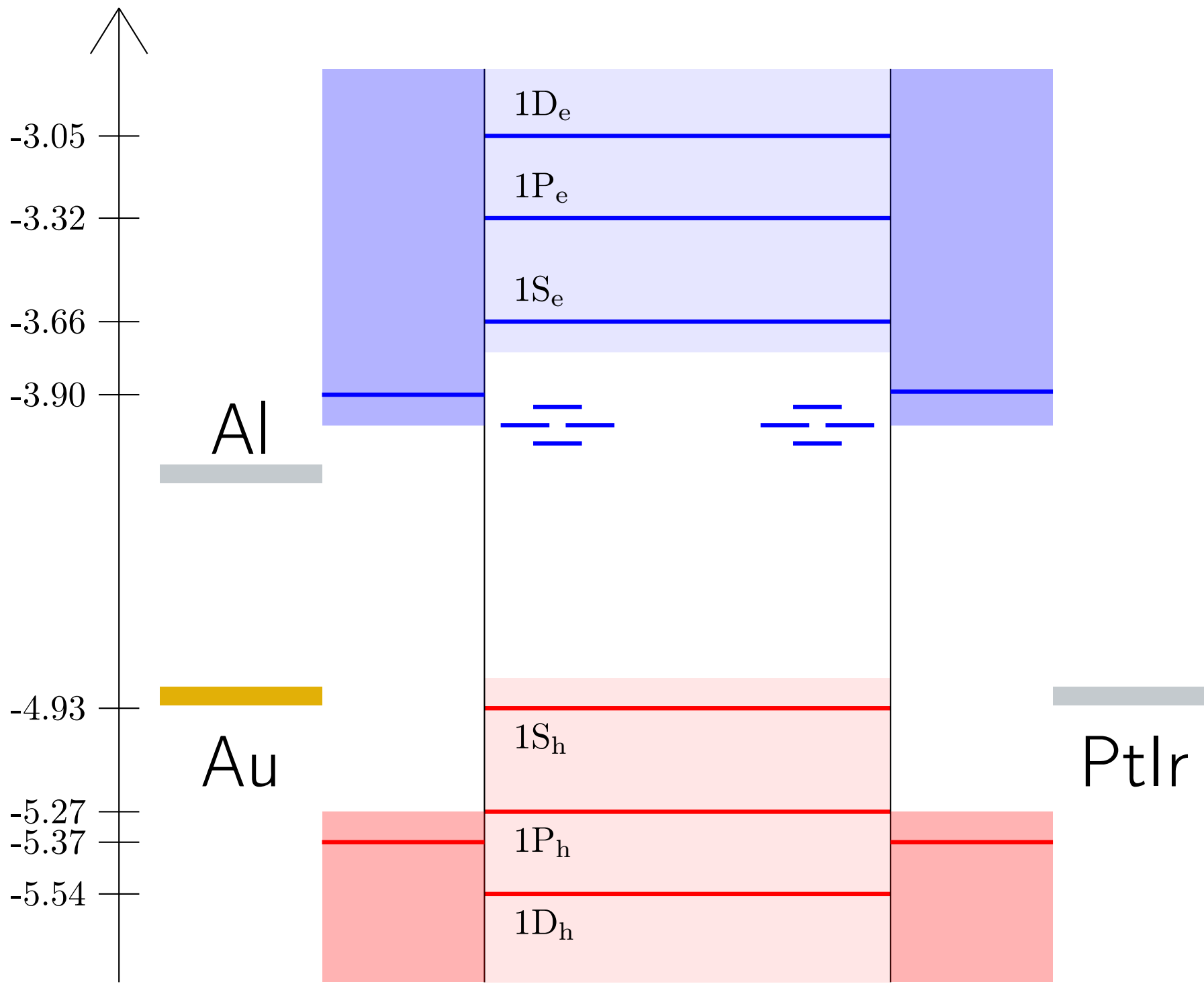

Figure S11: Sketch of the energy levels in PbS/$MAPbI_3$ QDs, corresponding to Table S1. The energy position relative to the vacuum level is taken from Piatkowski et al.[35] The work function of gold particles on a glass surface[38] and of the PtIr-coated silicon tip[39] are drawn at 4.9 eV. The work function of aluminum is drawn at 4.2 eV.[40]

function of the monolayer absorption. From the considerations of Piatkowski et al.[35] we know that for PbS QDs with a diameter of 3.2 nm in a $MAPbI_3$ matrix the conduction band minimum (CBM) of $MAPbI_3$ lies about 0.24 eV below the CBM of the PbS QDs and that the valence band maximum (VBM) of $MAPbI_3$ lies about 0.48 eV below the VBM of the PbS QDs. In addition, Jin et al.[36] showed experimentally that in PbS QDs a high density of trap states exists about 0.34 eV below the CBM. Further, Ning et al.[37] demonstrated by DFT calculations of PbS QDs in a $MAPbI_3$ matrix, that for mismatched QD/perovskite interfaces, trap states occur at the QD surface, predominantly close to the CBM. Summarizing these insights we arrive at the energy level diagram as plotted in Figure S11.

S5 Photocurrent AFM maps of a high N.A. focus on gold

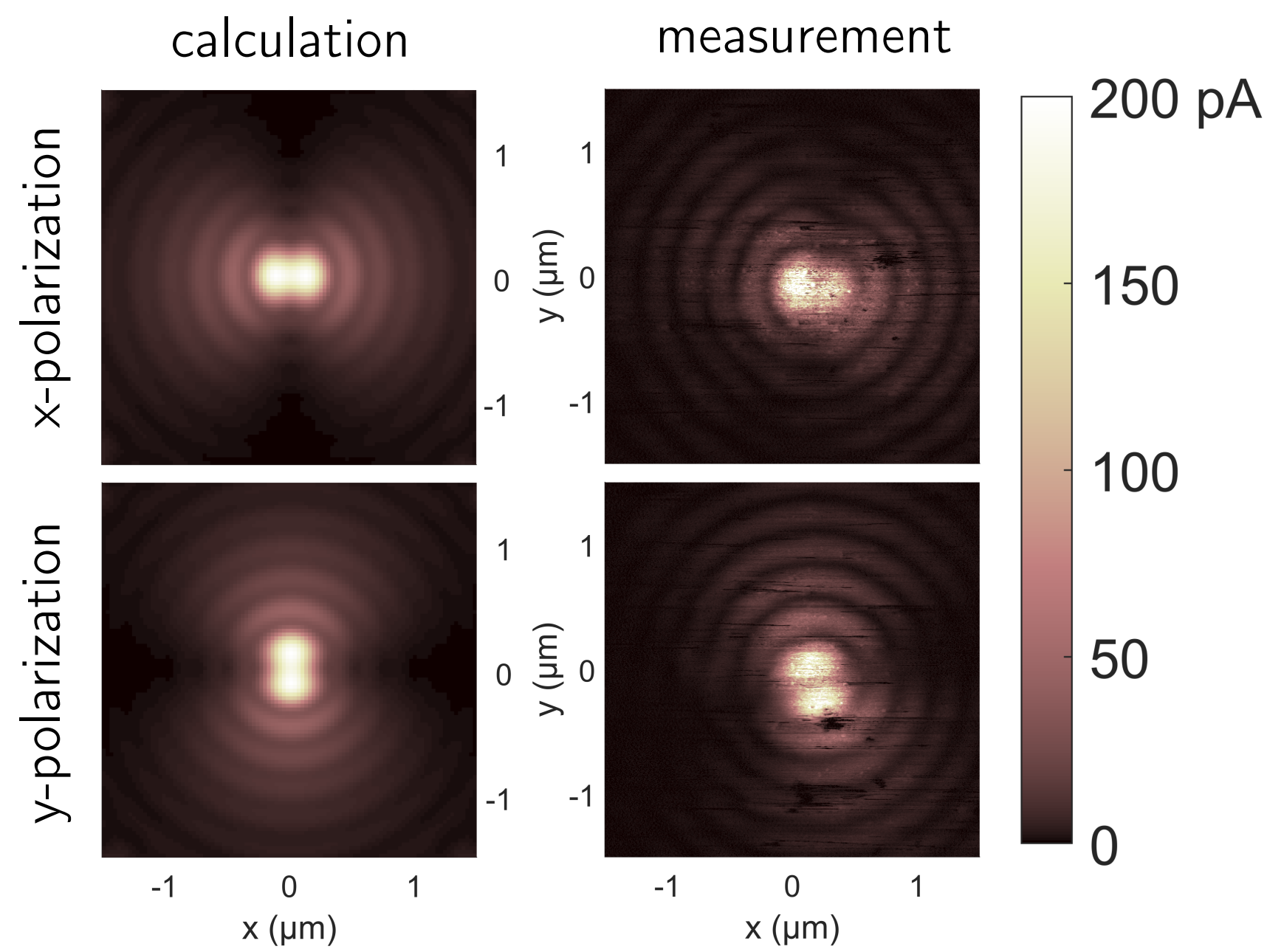

Figure S12: Photocurrent AFM maps of a 633 nm laser focused by a 1.45 N.A. objective (8 μW laser power, 300 mV bias, horizontal (x) and vertical (y) polarization) onto a 10 nm thick gold film covered by a PbS/$MAPbI_3$ QD monolayer (right column). The left column shows the numerically calculated patterns with intensity values scaled with a power law exponent of 0.64. This confirms that on the gold surfaces the spatial resolution is below 100 nm, just as on indium-tin-oxide-covered glass. [41]

S6 Bias variation for focus mapping on gold

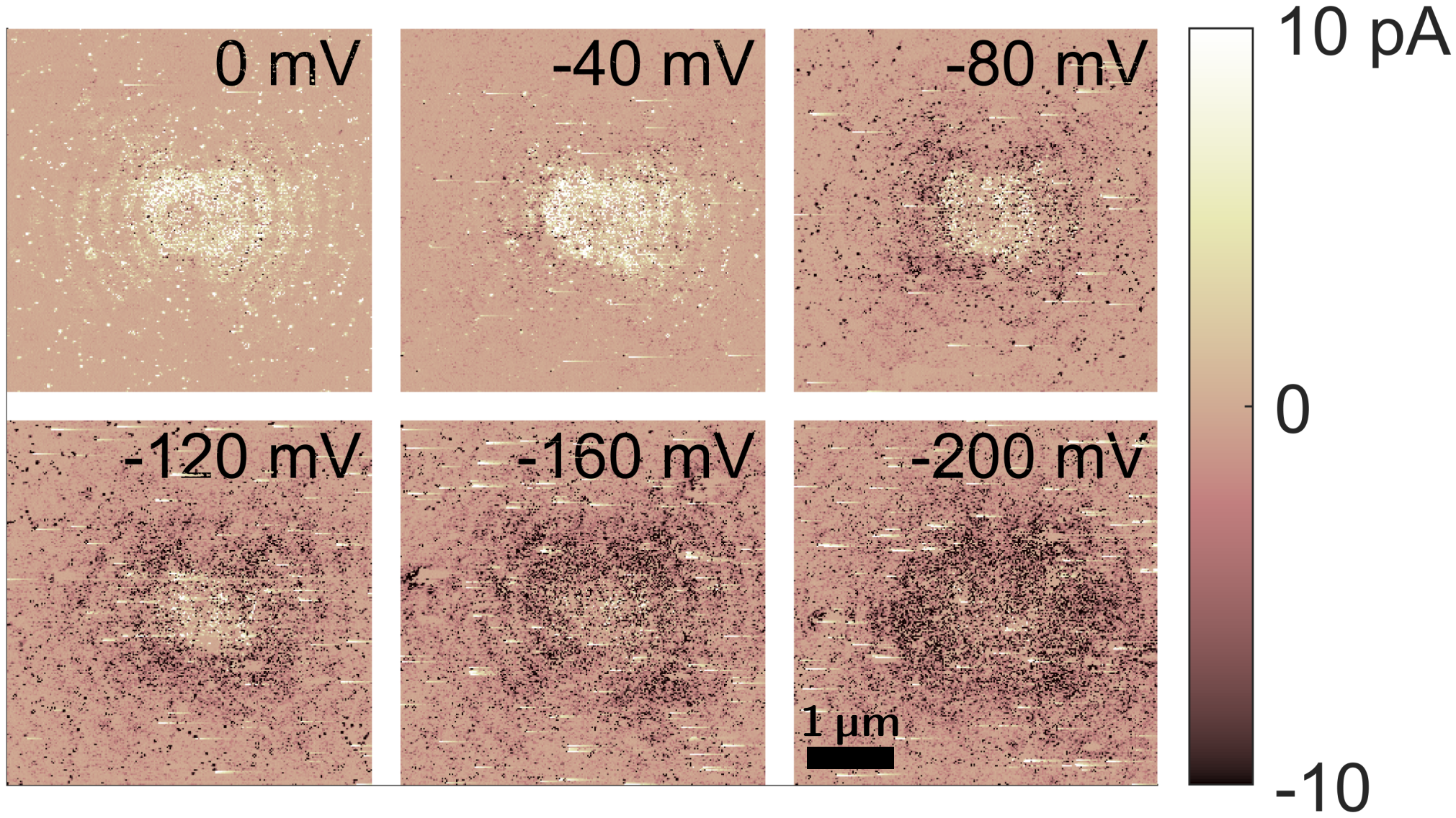

Figure S13: Bias-dependent photocurrent maps of the focus of a 1.45 N.A. objective (wavelength 633 nm, laser power 8 μW) on a flat 10 nm thick gold film that is covered with a QD monolayer. With increasing negative bias, it is first the low-intensity region (i.e., the outer part of the focus pattern) where the sign of the current switches from positive to negative. The high-intensity region (i.e., the inner part of the focus pattern) only follows at a higher bias. This demonstrates the different weight of, first, the bias-dependent current of photo-excited charges in the QD layer that depends sublinearly on intensity and, second, the bias-independent current of hot holes that follows the absorption in the gold film, depending linearly on intensity.

References

(1) Yang, Y.; Xie, H.; You, J.; Ye, W. Revisiting the Plasmon Radiation Damping of Gold Nanorods. *Phys. Chem. Chem. Phys.* **2022**, 4131–4135.

(2) Hohenester, U. *Nano and Quantum Optics - An Introduction to Basic Principles and Theory*; Springer Nature: Singapore, 2019.

(3) Foerster, B.; Rutten, J.; Pham, H.; Link, S.; Sönnichsen, C. Particle Plasmons as Dipole Antennas: State Representation of Relative Observables. *J. Phys. Chem. C* **2018**, *122*, 19116–19123.

(4) Kreibig, U. Small silver particles in photosensitive glass: Their nucleation and growth. *Appl. Phys.* **1976**, *10*, 255–264.

(5) Johnson, P. B.; Christy, R. W. Optical Constants of the Noble Metals. *Phys. Rev. B* **1972**, *6*, 4370–4379.

(6) Gao, H.; Li, K.; Kong, f.-m.; Xie, H.; Zhao, J. Optimizing Nano-Optical Antenna for the Enhancement of Spontaneous Emission. *Prog. Electromagn. Res.* **2010**, *104*, 313–331.

(7) Hartland, G. Optical Studies of Dynamics in Noble Metal Nanostructures. *Chem. Rev.* **2011**, *111*, 3858–3887.

(8) Sönnichsen, C.; Franzl, T.; Wilk, T.; Plessen, G.; Feldmann, J.; Wilson, O.; Mulvaney, P. Drastic Reduction of Plasmon Damping in Gold Nanorods. *Phys. Rev. Lett.* **2002**, *88*, 077402.

(9) Derkachova, A.; Kolwas, K.; Demchenko, I. Dielectric Function for Gold in Plasmonics Applications: Size Dependence of Plasmon Resonance Frequencies and Damping Rates for Nanospheres. *Plasmonics* **2016**, *11*, 941–951.

(10) Brown, A.; Sundararaman, R.; Narang, P.; Goddard, W.; Atwater, H. Non-Radiative Plasmon Decay and Hot Carrier Dynamics: Effects of Phonons, Surfaces and Geometry. *ACS Nano* **2016**, *10*, 957–966.

(11) Zolnai, Z.; Zambo, D.; Osváth, Z.; Nagy, N.; Fried, M.; Németh, A.; Pothorszky, S.; Deák, A. Gold Nanorod Plasmon Resonance Damping Effects on a Nanopatterned Substrate. *J. Phys. Chem. C* **2018**, *122*, 24941–24948.

(12) Foerster, B.; Joplin, A.; Kaefer, K.; Celiksoy, S.; Link, S.; Sönnichsen, C. Chemical Interface Damping Depends on Electrons Reaching the Surface. *ACS Nano* **2017**, *11*, 2886–2893.

(13) Lee, S.; Link, S. Chemical Interface Damping of Surface Plasmon Resonances. *Acc. Chem. Res.* **2021**, *54*, 1950–1960.

(14) Liu, M.; Pelton, M.; Guyot-Sionnest, P. Reduced damping of surface plasmons at low temperatures. *Phys. Rev. B* **2009**, *79*, 035418.

(15) Jain, P. K.; Lee, K. S.; El-Sayed, I. H.; El-Sayed, M. A. Calculated Absorption and Scattering Properties of Gold Nanoparticles of Different Size, Shape, and Composition: Applications in Biological Imaging and Biomedicine. *J. Phys. Chem. B* **2006**, *110*, 7238–7248.

(16) Colinge, J.-P.; Colinge, C. A. *Physics of Semiconductor Devices*, 1st ed.; Kluwer Academic Publishers: Dordrecht, 2002.

(17) Rhoderick, E.; Williams, R. *Metal-Semiconductor Contacts*, 2rd ed.; Oxford University Press: Oxford, 1988.

(18) Küstner, F.; Ditlbacher, H.; Hohenau, A.; Dirin, D.; Kovalenko, M.; Krenn, J. Quantitative photocurrent scanning probe microscopy on PbS quantum dot monolayers. *Nanoscale* **2024**, *16*, 16664–16670.

(19) Monch, W. On the physics of metal-semiconductor interfaces. *Rep. Prog. Phys.* **1990**, *53*, 221–278.

(20) Bardeen, J. Surface States and Rectification at a Metal Semi-Conductor Contact. *Phys. Rev.* **1947**, *71*, 717–727.

(21) Hasegawa, H. Fermi Level Pinning and Schottky Barrier Height Control at Metal-Semiconductor Interfaces of InP and Related Materials. *Jpn. J. Appl. Phys.* **1999**, *38*, 1098–1102.

(22) Mönch, W. Mechanisms of Schottky-barrier formation in metal–semiconductor contacts. *J. Vac. Sci. Technol. B* **1988**, *6*, 1270–1276.

(23) Tung, R. T. The physics and chemistry of the Schottky barrier height. *Appl. Phys. Rev.* **2014**, *1*, 011304.

(24) Koole, R.; Allan, G.; Delerue, C.; Meijerink, A.; Vanmaekelbergh, D.; Houtepen, A. J. Optical investigation of quantum confinement in PbSe nanocrystals at different points in the Brillouin zone. *Small* **2008**, *4*, 127–133.

(25) Moreels, I.; Zeger, H. On the Interpretation of Colloidal Quantum-Dot Absorption Spectra. *Small* **2008**, *4*, 1866–1868; author reply 1869.

(26) Cademartiri, L.; Montanari, E.; Calestani, G.; Migliori, A.; Guagliardi, A.; Ozin, G. Size-Dependent Extinction Coefficients of PbS Quantum Dots. *J. Am. Chem. Soc.* **2006**, *128*, 10337–10346.

(27) Sercel, P.; L. Lyons, J.; Bernstein, N.; Efros, A. Quasicubic model for metal halide perovskite nanocrystals. *J. Chem. Phys.* **2019**, *151*, 234106.

(28) Nanda, K.; Kruis, F.; Fissan, H.; Behera, S. Effective mass approximation for two extreme semiconductors: Band gap of PbS and CuBr nanoparticles. *J. Appl. Phys.* **2004**, *95*, 5035–5041.

(29) Kang, I.; Wise, F. Electronic structure and optical properties of PbS and PbSe quantum dots. *J. Opt. Soc. Am. B* **1997**, *14*, 1632–1646.

(30) Bera, D.; Qian, L.; Tseng, T.-K.; Holloway, P. H. Quantum Dots and Their Multimodal Applications: A Review. *Materials* **2010**, *3*, 2260 – 2345.

(31) Ma, Z.-Q.; Zhu, X.; Wang, C. Design of Lead Hybrid Halide Perovskite for Solar Cells. *J. Phys. Conf. Ser.* **2023**, *2473*, 012022.

(32) Phillips, L.; Rashed, A.; Treharne, R.; Kay, J.; Yates, P.; Mitrovic, I.; Weerakkody, A.; Hall, S.; Durose, K. Dispersion relation data for methylammonium lead triiodide perovskite deposited on a (100) silicon wafer using a two-step vapour-phase reaction process. *Data Br.* **2015**, *5*, 926–928.

(33) Rubino, A.; Caliò, L.; Calvo, M.; Míguez, H. Ligand-Free MAPbI 3 Quantum Dot Solar Cells based on Nanostructured Insulating Matrices. *Solar RRL* **2021**, *5*, 2100204.

(34) Enomoto, J.; Sato, R.; Yokoyama, M.; Kimura, T.; Oshita, N.; Umemoto, K.; Asakura, S.; Masuhara, A. Highly luminescent MAPbI 3 perovskite quantum dots with a simple purification process via ultrasound-assisted bead milling. *RSC Adv.* **2022**, *12*, 5571–5576.

(35) Piatkowski, P.; Masi, S.; Galar, P.; Gutiérrez Tovar, M.; Ngo, T. T.; Mora-Seró, I.; Douhal, A. Deciphering the Role of Quantum Dots Size in the Ultrafast Charge Carrier Dynamics at the Perovskite-Quantum Dots Interface. *J. Mater. Chem. C* **2020**, *8*, 14834–14844.

(36) Jin, Z.; Aiji, W.; Zhou, Q.; Wang, Y.; Wang, J. Detecting trap states in planar PbS colloidal quantum dot solar cells. *Sci. Rep.* **2016**, *6*, 37106.

(37) Ning, Z.; Gong, X.; Comin, R.; Walters, G.; Fan, F.; Voznyy, O.; Yassitepe, E.; Buin, A.;

Hoogland, S.; Sargent, E. H. Quantum-dot-in-perovskite solids. *Nature* **2015**, *523*, 324–328.

(38) Khabir, K. M.; Shahabuddin, M.; Noginova, N.; Noginov, M. Control of work functions of nanophotonic components. *Sci. Rep.* **2024**, *14*, 18044.

(39) Lv, Y.; Cui, J.; Jiang, Z. M.; Yang, X. Nanoscale electrical property studies of individual GeSi quantum rings by conductive scanning probe microscopy. *Nanoscale Res. Lett.* **2012**, *7*, 659.

(40) Kawano, H. Effective Work Functions of the Elements: Database, Most probable value, Previously recommended value, Polycrystalline thermionic contrast, Change at critical temperature, Anisotropic dependence sequence, Particle size dependence. *Prog. Surf. Sci.* **2022**, *97*, 100583.

(41) Küstner, F.; Hohenau, A.; Ditlbacher, H.; Krenn, J. R. Quantum-Dot-Mediated Light Field Imaging by Photocurrent Scanning Force Microscopy. *Nanophotonics* **2026**, *15*, e70057.
